# Supplementary material for: Plant P-bodies in post-transcriptional control: Composition, dynamics, and context-dependent roles
Source: Plant Commun. 2026 Mar 3;7(3):101787. doi: 10.1016/j.xplc.2026.101787 (PMC12983270; doi:10.1016/j.xplc.2026.101787)
Supplement: Document S1. Figure S1 [file mmc1.pdf]

Plant Communications, Volume *7*

Supplemental information

Plant P-bodies in post-transcriptional control: Composition, dynamics, and context-dependent roles

Arash Matinahmadi, Zoofa Zayani, Karolina Majewska, and Dariusz Jan Smoliński

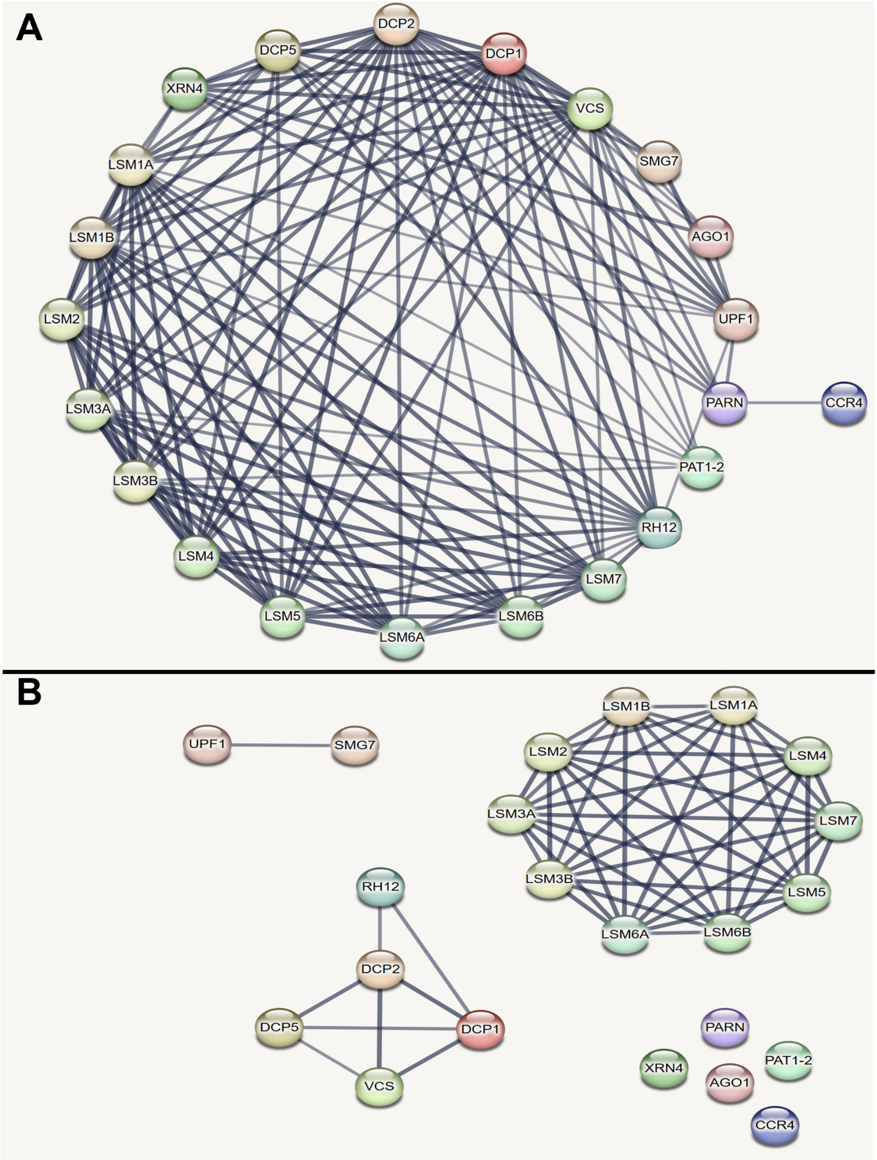

A
DCP2
DCP5
DCP1
XRN4
VCS
SMG7
LSM1A
LSM1B
AGO1
LSM2
UPF1
LSM3A
PARN
CCR4
PAT1-2
LSM3B
RH12
LSM4
LSM7
LSM5
LSM6B
LSM6A
B
LSM1B
LSM1A
UPF1
SMG7
LSM2
LSM4
LSM3A
LSM7
LSM3B
LSM5
RH12
LSM6A
LSM6B
DCP2
DCP5
DCP1
PARN
PAT1-2
XRN4
AGO1
VCS
CCR4
