# Supplementary material for: Plant P-bodies in post-transcriptional control: Composition, dynamics, and context-dependent roles
Source: Plant Commun. 2026 Mar 3;7(3):101787. doi: 10.1016/j.xplc.2026.101787 (PMC12983270; doi:10.1016/j.xplc.2026.101787)
Supplement: Table S1. Core PB-related factors across kingdoms — The aliases and functions have been adapted by the Tair (www.arabidopsis.org/), Saccharomyces Genome Database (https://www.yeastgenome.org/), UniProt (https://www.uniprot.org/), and NCBI (https://www.ncbi.nlm.nih.gov/) [file mmc2.pdf]

Supplemental Table 1 Core PB-Related Factors Across Kingdoms. The aliases and functions have been adapted by the Tair (www.arabidopsis.org/), Saccharomyces Genome Database (SGD) (https://www.yeastgenome.org/), UniProt (https://www.uniprot.org/), and NCBI (https://www.ncbi.nlm.nih.gov/).

Protein	Presence			Aliases			Function	IDR/IDP	References: Function IDR
	Plants	Yeast	Human	Plants	Yeast	Human			
DCP1 (mRNA-decapping enzyme subunit 1)	✓	✓	✓	–	–	DCP1A; DCP1B	Scaffold that recruits DCP2 and partners to assemble the decapping complex and stimulate mRNA decapping.	✓	(Xu et al., 2006; Sakuno et al., 2004; Nishimura et al., 2018; Panigrahi and Satapathy, 2020a; Jonas and Izaurraldes, 2013; Currie et al., 2023)
DCP2 (mRNA-decapping enzyme subunit 2)	✓	✓	✓	TRIDENT/TDT	–	NUDT20; NUDIX; HDpc	Catalytic decapping enzyme that removes the 5′ cap; localizes to PBs and acts with DCP1/DCP5. Activity is tightly controlled to avoid excessive decay.	✓	(Nishimura et al., 2018; Currie et al., 2023; Iwasaki et al., 2007; Van Dijk et al., 2002; Harigaya et al., 2010; Fromm et al., 2011; Wurm and Sprangers, 2019; Vukovic et al., 2024)
DCP5 (Protein Decapping 5)	✓	–	–	–	–	–	Plant-specific cofactor promoting DCP1-DCP2 and PB assembly; also acts nuclear role in modulating RNA Polymerase II enrichment at the FLC (Flowering Locus C), highlighting its involvement in transcriptional control. DCP5 has also been identified as a multifunctional osmosensor that participates in both osmosensing and osmotic stress adaptation.	✓	(Xu and Chua, 2011; Panigrahi and Satapathy, 2020a; Chantarachot and Bailey-Serres, 2018; Wang et al., 2024; Wang et al., 2023)
EDC3 (Enhancer of mRNA-decapping 3)	–	✓	✓	–	LSM16	LSM16; YJDC; YJEFN2; MRT50	Decapping activator that promotes efficient removal of the m7G cap.	✓	(Rao and Parker, 2017; Jonas and Izaurralde, 2013; Currie et al., 2023; Harigaya et al., 2010; Bearss et al., 2021)
EDC4 / VCS (Enhancer of mRNA-decapping 4)	✓	✓	✓	VARICOSE (VCS)	EDC4L; Pdc1	Ge-1; RCD-8; HEDLS	Scaffold that binds DCP1, enhances DCP2, and recruits mRNA/cofactors to assemble the decapping complex.	✓	(Xu et al., 2006; Jonas and Izaurralde, 2013; Chantarachot and Bailey-Serres, 2018; Seto et al., 2015; Ling et al., 2014; Panigrahi et al.,2024)
XRN1 (5′→3′ exoribonuclease 1)	–	✓	✓	–	DST2; KEM1; RAR5; SEP1; SKI1	SEP1	Major 5′→3′ exonuclease degrading uncapped transcripts.	✓	(Eulalio et al., 2007a; Jonas and Izaurralde, 2013; Kulkarni et al., 2010; Vaškovičová et al., 2017; Brothers et al., 2023)

XRN4 (5′→3′ exoribonuclease 4)	✓	–	–	AIN1; EIN5	–	–	Plant homolog of XRN1; key for mRNA turnover and stress-regulated decay; often in PBs; can act co-translationally.	✓	(Weber et al., 2008; Maldonado-Bonilla., 2014; Eulalio et al., 2007a; Jang et al., 2020; Rymarquis et al., 2011; Song et al., 2021; Carpentier et al., 2020)<br><br>IDR: Uniport ID Q9FQ04
LSM1–7 (Sm-like proteins)	✓	✓	✓	LSM1A/B; LSM3A/B; LSM5 (SAD1); LSM7 (EMB2816)	Lsm1 (SPB8); Lsm2 (SMX5, SNP3); Lsm3 (SMX4, USS2); Lsm4 (SDB23, USS1)	Lsm1 (CASM); Lsm2 (G7B); Lsm4 (GRP)	Heptamer that binds oligoadenylated 3′ ends, recruits decapping factors to PBs, and aids stress-induced decay.	✓	(Currie et al., 2023; Chantarachot and Bailey-Serres., 2018;Mazzoni et al., 2007; Chowdhury et al., 2007; Golisz et al., 2013; Perea-Resa et al., 2016; Protter et al., 2018)
PAT1 (Protein associated with topoisomerase I-decapping regulator)	✓	✓	✓	PAT1H; MRT1	Pat1	PATL1	Translation repressor forming the LSM1-7-PAT1 complex; promotes decapping of oligoadenylated mRNAs in PBs.	✓	(Jonas and Izaurralde., 2013; Currie et al., 2023; Scheller et al., 2007; Haas et al., 2010; Sharif and Conti, 2013; Pilkington and Parker, 2008; Marnef and Standart, 2010; Roux et al., 2015; Zuo et al., 2021)
Dhh1 / DDX6 (DEAD-box helicases)	✓	✓	✓	RH6; RH8; RH12; RH14	Dhh1	RCK / p54	Helicases mediating translational repression and decay; stabilize PBs/SGs and cooperate with LSM1-7-PAT1.	✓	(Rao and Parker, 2017; Jonas and Izaurralde, 2013; Currie et al., 2023; Chantarachot and Bailey-Serres., 2018; Chantarachot et al., 2020; Bhullar et al., 2017; Sharif et al., 2013; Coller et al., 2001; Mugler et al., 2016; Presnyak and Coller, 2013; Zuo et al., 2024; Kamenska et al., 2016)
CCR4–NOT (deadenylase complex)	✓	✓	✓	—	FUN27; NUT21; CNOT1	CNOT1	Deadenylation via CCR4/CAF1 under NOT control; interfaces with PAT1 and DDX6/Dhh1 in decay.	✓	(Maldonado-Bonilla, 2014; Mugler et al., 2016; Zhang et al., 2020; Mittal et al., 2011; Collart and Panasenko, 2012; Abbasi et al., 2013; Fang et al., 2021; Ito et al., 2011)
PARN (poly(A)-specific ribonuclease)	✓	–	✓	AHG2	–	CSP9; EIF2C	Context-dependent deadenylase acting alone or with CCR4-NOT, especially under stress or DNA damage.	✓	(Maldonado-Bonilla, 2014; Aizer et al., 2014; Hirayama, 2021; Panigrahi and Satapathy, 2020b;

									Moreno et al, 2013; Duan et al., 2019)
AGO1 (Argonaute 1)	✓	✓	✓	—	—	—	RISC core; small-RNA-guided cleavage or translational repression.	✓	(Liu et al., 2005; Rossi, 2005; Jabri, 2005; Vaucheret, 2008; Pomeranz et al., 2010a; Blagojevic et al., 2024)
GW182 / TNRC6	–	✓	✓	—	TNRC6	TNRC6; KIAA1460; FAM E6; EDIE	Scaffold partnering with Argonautes and recruiting CCR4-NOT/PAN deadenylases.	✓	(Behm-Ansmant et al., 2006a; Behm-Ansmant et al., 2006b; Sheu-Gruttadauria and MacRae, 2018)
UPF1 (Up-frameshift 1)	✓	✓	✓	ATUPF1; LBA1; RENT1	NAM7; IFS2; MOF4; SUT2; SUP113	RENT1; NORF1; KIAA0221	Master NMD helicase; in plants partners with SMG7 and can route Premature Termination Codon (PTC) transcripts to PBs.	✓	(Chantarachot and Bailey-Serres., 2018; Chicois et al., 2018; Brogna et al., 2008; Sheth and Parker., 2006; Dai et al., 2016; Van Der Lee et al., 2014)
SMG7	✓	✓	✓	F7K24	—	EST1C; KIAA0250	NMD effector binding phosphorylated UPF1; promotes PB relocalization and recruits CCR4-NOT.	✓	(Eulalio et al., 2007a; Chantarachot and Bailey-Serres, 2018; Nicholson and Mühlemann, 2010; Capitao et al., 2018; Mérai et al., 2013; Riehs et al., 2008)
eIF4E (cap-binding factor)	–	✓	✓	—	CDC33; TIF45	eIF4E1	Cap-binding initiation factor that promotes ribosome recruitment and 5′ UTR unwinding.	-	(Xu and Chua, 2011; Parker and Sheth, 2007; Chantarachot and Bailey-Serres, 2018; Brengues and Parker, 2007)
SKI2 (SKI complex helicase)	–	✓	✓	—	SKI2	SKIV2L; DDX13	Helicase of the SKI-exosome pathway aiding RNA decay and quality control.	✓	(Chicois et al., 2018; Huch and Nissan, 2017; Halbach et al., 2012; Li et al., 2023; Zhao and Kunst, 2016)

Supplemental References

Abbasi, N., Park, Y. I., & Choi, S. B. (2013). RNA deadenylation and decay in plants. *Journal of Plant Biology*, *56*(4), 198-207.

Aizer, A., Kalo, A., Kafri, P., Shraga, A., Ben-Yishay, R., Jacob, A., Kinor, N. & Shav-Tal, Y. (2014). Quantifying mRNA targeting to P-bodies in living human cells reveals their dual role in mRNA decay and storage. *Journal of cell science*, *127*(20), 4443-4456.

Bearss J.J., Padi S.K., Singh N., (2021) EDC3 phosphorylation regulates growth and invasion through controlling P-body formation and dynamics *EMBO Rep.* 22(4):e50835. doi: 10.15252/embr.202050835.

Behm-Ansmant, I., Rehwinkel, J., & Izaurralde, E. (2006). MicroRNAs silence gene expression by repressing protein expression and/or by promoting mRNA decay. In *Cold Spring Harbor symposia on quantitative biology* 71, 523-530.

Behm-Ansmant, I., Rehwinkel, J., Doerks, T., Stark, A., Bork, P., & Izaurralde, E. (2006). mRNA degradation by miRNAs and GW182 requires both CCR4: NOT deadenylase and DCP1: DCP2 decapping complexes. *Genes & development*, *20*(14), 1885-1898.

Bhullar, D. S., Sheahan, M. B., & Rose, R. J. (2017). RNA processing body (P-body) dynamics in mesophyll protoplasts re-initiating cell division. *Protoplasma*, *254*(4), 1627-1637.

Blagojevic, A., Baldrich, P., Schiaffini, M., Lechner, E., Baumberger, N., Hammann, P., Elmayan, T., Garcia, D., Vaucheret, H., Meyers, B.C. and Genschik, P. (2024). Heat stress promotes Arabidopsis AGO1 phase separation and association with stress granule components. *Iscience*, *27*(3).

Brengues, M., & Parker, R. (2007). Accumulation of polyadenylated mRNA, Pab1p, eIF4E, and eIF4G with P-bodies in Saccharomyces cerevisiae. *Molecular biology of the cell*, *18*(7), 2592-2602.

Brogna, S., Ramanathan, P., & Wen, J. (2008). UPF1 P-body localization. *Biochemical Society Transactions*, *36*(4), 698-700.

Brothers, W. R., Ali, F., Kajjo, S., & Fabian, M. R. (2023). The EDC4-XRN1 interaction controls P-body dynamics to link mRNA decapping with decay. *The EMBO Journal*, *42*(21), EMBJ 2023 113933.

Capitao, C., Shukla, N., Wandrolova, A., Mittelsten Scheid, O., & Riha, K. (2018). Functional characterization of SMG7 paralogs in Arabidopsis thaliana. *Frontiers in Plant Science*, *9*, 1602.

Carpentier, M. C., Deragon, J. M., Jean, V., Be, S. H. V., Bousquet-Antonelli, C., & Merret, R. (2020). Monitoring of XRN4 targets reveals the importance of cotranslational decay during Arabidopsis development. *Plant Physiology*, *184*(3), 1251-1262.

Chantarachot, T., & Bailey-Serres, J. (2018). Polysomes, stress granules, and processing bodies: a dynamic triumvirate controlling cytoplasmic mRNA fate and function. *Plant physiology*, *176*(1), 254-269.

Chantarachot, T., Sorenson, R.S., Hummel, M., Ke, H., Kettenburg, A.T., Chen, D., Aiyetiwa, K., Dehesh, K., Eulgem, T., Sieburth, L.E. and Bailey-Serres, J. (2020). DHH1/DDX6-like RNA helicases maintain ephemeral half-lives of stress-response mRNAs. *Nature Plants*, *6*(6), 675-685.

Chicois, C., Scheer, H., Garcia, S., Zuber, H., Mutterer, J., Chicher, J., Hammann, P., Gagliardi, D. and Garcia, D. (2018). The UPF1 interactome reveals interaction networks between RNA degradation and translation repression factors in Arabidopsis. *The Plant*

Chowdhury, A., Mukhopadhyay, J., & Tharun, S. (2007). The decapping activator Lsm1p-7p–Pat1p complex has the intrinsic ability to distinguish between oligoadenylated and polyadenylated RNAs. *Rna*, *13*(7), 998-1016.

Collart, M. A., & Panasenko, O. O. (2012). The Ccr4–not complex. *Gene*, *492*(1), 42-53.

Coller, J. M., Tucker, M., Sheth, U., Valencia-Sanchez, M. A., & Parker, R. (2001). The DEAD box helicase, Dhh1p, functions in mRNA decapping and interacts with both the decapping and deadenylase complexes. *Rna*, *7*(12), 1717-1727.

Currie, S. L., Xing, W., Muhlrad, D., Decker, C. J., Parker, R., & Rosen, M. K. (2023). Quantitative reconstitution of yeast RNA processing bodies. *Proceedings of the National Academy of Sciences*, *120*(14), e2214064120.

Dai, Y., Li, W., & An, L. (2016). NMD mechanism and the functions of Upf proteins in plant. *Plant cell reports*, *35*(1), 5-15.

Duan, T. L., He, G. J., Hu, L. D., & Yan, Y. B. (2019). The intrinsically disordered C-terminal domain triggers nucleolar localization and function switch of PARN in response to DNA damage. *Cells*, *8*(8), 836.

Eulalio, A., Behm-Ansmant, I., & Izaurralde, E. (2007a). P bodies: at the crossroads of post-transcriptional pathways. *Nature reviews Molecular cell biology*, *8*(1), 9-22.

Fang, J. C., Tsai, Y. C., Chou, W. L., Liu, H. Y., Chang, C. C., Wu, S. J., & Lu, C. A. (2021). A CCR4-associated factor 1, OsCAF1B, confers tolerance of low-temperature stress to rice seedlings. *Plant Molecular Biology*, *105*(1), 177-192.

Fromm, S. A., Truffault, V., Kamenz, J., Braun, J. E., Hoffmann, N. A., Izaurralde, E., & Sprangers, R. (2011). The structural basis of Edc3-and Scd6-mediated activation of the Dcp1: Dcp2 mRNA decapping complex. *The EMBO journal*, *31*(2), 279.

Golisz, A., Sikorski, P. J., Kruszka, K., & Kufel, J. (2013). Arabidopsis thaliana LSM proteins function in mRNA splicing and degradation. *Nucleic acids research*, *41*(12), 6232-6249.

Haas, G., Braun, J. E., Igreja, C., Tritschler, F., Nishihara, T., & Izaurralde, E. (2010). HPat provides a link between deadenylation and decapping in metazoa. *Journal of Cell Biology*, *189*(2), 289-302.

Halbach, F., Rode, M., & Conti, E. (2012). The crystal structure of S. cerevisiae Ski2, a DExH helicase associated with the cytoplasmic functions of the exosome. *Rna*, *18*(1), 124-134.

Harigaya, Y., Jones, B. N., Muhlrad, D., Gross, J. D., & Parker, R. (2010). Identification and analysis of the interaction between Edc3 and Dcp2 in Saccharomyces cerevisiae. *Molecular and cellular biology*, *30*(6), 1446-1456.

Hirayama, T. (2021). PARN-like Proteins regulate gene expression in land plant mitochondria by modulating mRNA polyadenylation. *International journal of molecular sciences*, *22*(19), 10776.

Huch, S., & Nissan, T. (2017). An mRNA decapping mutant deficient in P body assembly limits mRNA stabilization in response to osmotic stress. *Scientific reports*, *7*(1), 44395.

Ito, K., Takahashi, A., Morita, M., Suzuki, T., & Yamamoto, T. (2011). The role of the CNOT1 subunit of the CCR4-NOT complex in mRNA deadenylation and cell viability. *Protein & cell*, *2*(9), 755-763.

Iwasaki, S., Takeda, A., Motose, H., & Watanabe, Y. (2007). Characterization of Arabidopsis decapping proteins AtDCP1 and AtDCP2, which are essential for post-embryonic development. *FEBS letters*, *581*(13), 2455-2459.

Jabri, E. (2005). P-bodies take a RISC. *Nature structural & molecular biology*, *12*(7), 564-564.

Jang, G. J., Jang, J. C., & Wu, S. H. (2020). Dynamics and functions of stress granules and processing bodies in plants. *Plants*, *9*(9), 1122.

Jonas, S., & Izaurralde, E. (2013). The role of disordered protein regions in the assembly of decapping complexes and RNP granules. *Genes & development*, *27*(24), 2628-2641.

Kamenska, A., Simpson, C., Vindry, C., Broomhead, H., Bénard, M., Ernoult-Lange, M., Lee, B.P., Harries, L.W., Weil, D. and Standart, N. (2016). The DDX6–4E-T interaction mediates translational repression and P-body assembly. *Nucleic acids research*, *44*(13), 6318-6334.

Kulkarni, M., Ozgur, S., & Stoecklin, G. (2010). On track with P-bodies. *Biochemical Society Transactions*, *38*(1), 242-251.

Li, X., Li, C., Zhu, J., Zhong, S., Zhu, H., & Zhang, X. (2023). Functions and mechanisms of RNA helicases in plants. *Journal of Experimental Botany*, *74*(7), 2295-2310.

Ling, Y. H., Wong, C. C., Li, K. W., Chan, K. M., Boukamp, P., & Liu, W. K. (2014). CCHCR1 interacts with EDC4, suggesting its localization in P-bodies. *Experimental cell research*, *327*(1), 12-23.

Liu, J., Valencia-Sanchez, M. A., Hannon, G. J., & Parker, R. (2005). MicroRNA-dependent localization of targeted mRNAs to mammalian P-bodies. *Nature cell biology*, *7*(7), 719-723.

Maldonado-Bonilla, L. D. (2014). Composition and function of P bodies in Arabidopsis thaliana. *Frontiers in plant science*, *5*, 201.

Marnef, A., & Standart, N. (2010). Pat1 proteins: a life in translation, translation repression and mRNA decay. *Biochemical Society Transactions*, *38*(6), 1602-1607.

Mazzoni, C., D'Addario, I., & Falcone, C. (2007). The C-terminus of the yeast Lsm4p is required for the association to P-bodies. *FEBS letters*, *581*(25), 4836-4840.

Mérai, Z., Benkovics, A.H., Nyikó, T., Debreczeny, M., Hiripi, L., Kerényi, Z., Kondorosi, É. and Silhavy, D. (2013). The late steps of plant nonsense-mediated mRNA decay. *The Plant Journal*, *73*(1), 50-62.

Mittal, S., Aslam, A., Doidge, R., Medica, R., & Winkler, G. S. (2011). The Ccr4a (CNOT6) and Ccr4b (CNOT6L) deadenylase subunits of the human Ccr4–Not complex contribute to the prevention of cell death and senescence. *Molecular biology of the cell*, *22*(6), 748-758.

Moreno, A. B., Martínez de Alba, A. E., Bardou, F., Crespi, M. D., Vaucheret, H., Maizel, A., & Mallory, A. C. (2013). Cytoplasmic and nuclear quality control and turnover of single-stranded RNA modulate post-transcriptional gene silencing in plants. *Nucleic Acids Research*, *41*(8), 4699-4708.

Mugler, C.F., Hondele, M., Heinrich, S., Sachdev, R., Vallotton, P., Koek, A.Y., Chan, L.Y. and Weis, K. (2016). ATPase activity of the DEAD-box protein Dhh1 controls processing body formation. *elife*, *5*, e18746.

Nicholson, P., & Mühlemann, O. (2010). Cutting the nonsense: the degradation of PTC-containing mRNAs. *Biochemical Society Transactions*, *38*(6), 1615-1620.

Nishimura, T., Fakim, H., Brandmann, T., Youn, J. Y., Gingras, A. C., Jinek, M., & Fabian, M. R. (2018). Human MARF1 is an endoribonuclease that interacts with the DCP1: 2 decapping complex and degrades target mRNAs. *Nucleic acids research*, *46*(22), 12008-12021.

Panigrahi, G. K., & Satapathy, K. B. (2020a). Arabidopsis DCP5, a decapping complex protein interacts with ubiquitin-5 in the processing bodies. *Plant Archives (09725210)*, *20*(1).

Panigrahi, G. K., & Satapathy, K. B. (2020b). Formation of Arabidopsis poly (A)-specific ribonuclease associated processing bodies in response to pathogenic infection, *Plant Archives*, 20(2), 4907-4912.

Panigrahi, G. K., Sahoo, A., & Satapathy, K. B. (2024). The processing body component varicose plays a multiplayer role towards stress management in Arabidopsis. *Plant Physiology Reports*, *29*(1), 186-192.

Parker, R., & Sheth, U. (2007). P bodies and the control of mRNA translation and degradation. *Molecular cell*, *25*(5), 635-646.

Perea-Resa, C., Carrasco-López, C., Catalá, R., Turečková, V., Novak, O., Zhang, W., Sieburth, L., Jiménez-Gómez, J.M. and Salinas, J. (2016). The LSM1-7 complex differentially regulates Arabidopsis tolerance to abiotic stress conditions by promoting selective mRNA decapping. *The Plant Cell*, *28*(2), 505-520.

Pilkington, G. R., & Parker, R. (2008). Pat1 contains distinct functional domains that promote P-body assembly and activation of decapping. *Molecular and cellular biology*, *28*(4), 1298-1312.

Pomeranz, M. C., Hah, C., Lin, P. C., Kang, S. G., Finer, J. J., Blackshear, P. J., & Jang, J. C. (2010a). The Arabidopsis tandem zinc finger protein AtTZF1 traffics between the nucleus and cytoplasmic foci and binds both DNA and RNA. *Plant physiology*, *152*(1), 151-165.

Presnyak, V., & Coller, J. (2013). The DHH1/RCKp54 family of helicases: an ancient family of proteins that promote translational silencing. *Biochimica et Biophysica Acta (BBA)-Gene Regulatory Mechanisms*, *1829*(8), 817-823.

Protter, D. S., Rao, B. S., Van Treeck, B., Lin, Y., Mizoue, L., Rosen, M. K., & Parker, R. (2018). Intrinsically disordered regions can contribute promiscuous interactions to RNP granule assembly. *Cell reports*, *22*(6), 1401-1412.

Rao, B. S., & Parker, R. (2017). Numerous interactions act redundantly to assemble a tunable size of P bodies in Saccharomyces cerevisiae. *Proceedings of the National Academy of Sciences*, *114*(45), E9569-E9578.

Riehs, N., Akimcheva, S., Puizina, J., Bulankova, P., Idol, R.A., Siroky, J., Schleiffer, A., Schweizer, D., Shippen, D.E. and Riha, K. (2008). Arabidopsis SMG7 protein is required for exit from meiosis. *Journal of cell science*, *121*(13), 2208-2216.

Rossi, J. J. (2005). RNAi and the P-body connection. *Nature Cell Biology*, *7*(7), 643-644.

Roux, M.E., Rasmussen, M.W., Palma, K., Lolle, S., Regué, À.M., Bethke, G., Glazebrook, J., Zhang, W., Sieburth, L., Larsen, M.R. and Mundy, J. (2015). The mRNA decay factor PAT1 functions in a pathway including MAP kinase 4 and immune receptor SUMM2. *The EMBO Journal*, *34*(5), 593-608.

Rymarquis, L. A., Souret, F. F., & Green, P. J. (2011). Evidence that XRN4, an Arabidopsis homolog of exoribonuclease XRN1, preferentially impacts transcripts with certain sequences or in particular functional categories. *Rna*, *17*(3), 501-511.

Sakuno T., Araki Y., Ohya Y., (2004) Decapping reaction of mRNA requires Dcp1 in fission yeast: its characterization in different species from yeast to human *J. Biochem.* 136(6), 805-812

cheller, N., Resa-Infante, P., de la Luna, S., Galao, R.P., Albrecht, M., Kaestner, L., Lipp, P., Lengauer, T., Meyerhans, A. and Díez, J. (2007). Identification of PatL1, a human

homolog to yeast P body component Pat1. *Biochimica Et Biophysica Acta (BBA)-Molecular Cell Research*, *1773*(12), 1786-1792.

Seto, E., Yoshida-Sugitani, R., Kobayashi, T., & Toyama-Sorimachi, N. (2015). The assembly of EDC4 and Dcp1a into processing bodies is critical for the translational regulation of IL-6. *PloS one*, *10*(5), e0123223.

Sharif, H., & Conti, E. (2013). Architecture of the Lsm1-7-Pat1 complex: a conserved assembly in eukaryotic mRNA turnover. *Cell reports*, *5*(2), 283-291.

Sharif, H., Ozgur, S., Sharma, K., Basquin, C., Urlaub, H., & Conti, E. (2013). Structural analysis of the yeast Dhh1–Pat1 complex reveals how Dhh1 engages Pat1, Edc3 and RNA in mutually exclusive interactions. *Nucleic acids research*, *41*(17), 8377-8390.

Sheth, U., & Parker, R. (2006). Targeting of aberrant mRNAs to cytoplasmic processing bodies. *Cell*, *125*(6), 1095-1109.

Sheu-Gruttadauria, J., & MacRae, I. J. (2018). Phase transitions in the assembly and function of human miRISC. *Cell*, *173*(4), 946-957.

Song, W., Li, Y., Niu, Y., Wu, Y., Bao, Y., & Yu, X. (2021). Global characterization of XRN 5′-3′ exoribonucleases and their responses to environmental stresses in plants. *Diversity*, *13*(12), 612.

Van Der Lee, R., Buljan, M., Lang, B., Weatheritt, R.J., Daughdrill, G.W., Dunker, A.K., Fuxreiter, M., Gough, J., Gsponer, J., Jones, D.T. and Kim, P.M. (2014). Classification of intrinsically disordered regions and proteins. *Chemical reviews*, *114*(13), 6589-6631.

Van Dijk, E., Cougot, N., Meyer, S., Babajko, S., Wahle, E., & Séraphin, B. (2002). Human Dcp2: a catalytically active mRNA decapping enzyme located in specific cytoplasmic structures. *The EMBO journal*, *21*(24), 6915-6924.

Vaškovičová, K., Awadová, T., Veselá, P., Balážová, M., Opekarová, M., & Malinsky, J. (2017). mRNA decay is regulated via sequestration of the conserved 5′-3′ exoribonuclease Xrn1 at eisosome in yeast. *European journal of cell biology*, *96*(6), 591-599.

Vaucheret, H. (2008). Plant argonautes. *Trends in plant science*, *13*(7), 350-358.

Vukovic, I., Barnada, S. M., Ruffin, J. W., Karlin, J., Lokareddy, R. K., Cingolani, G., & McMahon, S. B. (2024). Non-redundant roles for the human mRNA decapping cofactor paralogs DCP1a and DCP1b. *Life Science Alliance*, *7*(11).

Wang, W., Wang, C., Wang, Y., Ma, J., Wang, T., Tao, Z., Liu, P., Li, S., Hu, Y., Gu, A. and Wang, H. (2023). The P-body component DECAPPING5 and the floral repressor SISTER OF FCA regulate FLOWERING LOCUS C transcription in Arabidopsis. *The Plant Cell*, *35*(9), 3303-3324.

Wang, Z., Yang, Q., Zhang, D., Lu, Y., Wang, Y., Pan, Y., Qiu, Y., Men, Y., Yan, W., Xiao, Z. and Sun, R. (2024). A cytoplasmic osmosensing mechanism mediated by molecular crowding–sensitive DCP5. *Science*, *386*(6721), eadk9067.

Weber, C., Nover, L., & Fauth, M. (2008). Plant stress granules and mRNA processing bodies are distinct from heat stress granules. *The Plant Journal*, *56*(4), 517-530.

Wurm, J. P., & Sprangers, R. (2019). Dcp2: an mRNA decapping enzyme that adopts many different shapes and forms. *Current opinion in structural biology*, *59*, 115-123.

Xu, J., & Chua, N. H. (2009). Arabidopsis decapping 5 is required for mRNA decapping, P-body formation, and translational repression during postembryonic development. *The Plant Cell*, *21*(10), 3270-3279.

Xu, J., & Chua, N. H. (2011). Processing bodies and plant development. *Current opinion in plant biology*, *14*(1), 88-93.

Xu, J., Yang, J. Y., Niu, Q. W., & Chua, N. H. (2006). Arabidopsis DCP2, DCP1, and VARICOSE form a decapping complex required for postembryonic development. *The Plant Cell*, *18*(12), 3386-3398.

Zhang, Z.J., Gao, Q., Fang, X.D., Ding, Z.H., Gao, D.M., Xu, W.Y., Cao, Q., Qiao, J.H., Yang, Y.Z., Han, C. and Wang, Y. (2020). CCR4, a RNA decay factor, is hijacked by a plant cytorhabdovirus phosphoprotein to facilitate virus replication. *Elife*, *9*, e53753.

Zhao, L., & Kunst, L. (2016). SUPERKILLER complex components are required for the RNA exosome-mediated control of cuticular wax biosynthesis in Arabidopsis inflorescence stems. *Plant physiology*, *171*(2), 960-973.

Zuo, Z., Roux, M. E., Dagdas, Y. F., Rodriguez, E., & Petersen, M. (2024). PAT m RNA decapping factors are required for proper development in Arabidopsis. *FEBS lett.*, *598*(9), 1008-1021.

Zuo Z., Roux M.E., Sæmundsson H.P., Müller M., Munne Bosch S., Petersen M., (2021) The Arabidopsis thaliana mRNA decay factor PAT1 functions in osmotic stress responses and decaps ABA-responsive genes *FEBS Lett*. 595(2), 253-263.
