# Supplementary material for: Plant P-bodies in post-transcriptional control: Composition, dynamics, and context-dependent roles
Source: Plant Commun. 2026 Mar 3;7(3):101787. doi: 10.1016/j.xplc.2026.101787 (PMC12983270; doi:10.1016/j.xplc.2026.101787)
Supplement: Document S2. Article plus supplemental information [file mmc3.pdf]

Plant Communications
Review article

CellPress Partner Journal

Plant P-bodies in post-transcriptional control: Composition, dynamics, and context-dependent roles

Arash Matinahmadi[1,*], Zoofa Zayani[2], Karolina Majewska[1,2] and Dariusz Jan Smoliński[1,2,*]

[1]Department of Cellular and Molecular Biology, Nicolaus Copernicus University, 87-100 Torun, Poland

[2]Institute of Advanced Studies, Nicolaus Copernicus University, 87-100 Torun, Poland

***Correspondence: Arash Matinahmadi (arash.matinahmadi@doktorant.umk.pl), Dariusz Jan Smoliński (darsmol@umk.pl)**

https://doi.org/10.1016/j.xplc.2026.101787

ABSTRACT

Processing bodies (P-bodies, PBs) are cytoplasmic ribonucleoprotein condensates that concentrate mRNA-decay and translation-repression factors. In plants, PBs share core machinery with other eukaryotes but exhibit unique, context-dependent features that distinguish them from their yeast and mammalian counterparts. These properties are shaped by direct modulation from hormonal signaling (e.g., abscisic acid [ABA]) and stress physiology, underscoring their specialized roles in adaptation. Here we synthesize plant-focused evidence on PB composition, liquid–liquid phase separation (LLPS)-driven assembly, and their coupling to decapping-dependent and co-translational decay pathways. We delineate the contexts in which PBs act as decay hotspots versus buffering sites for non-translating mRNAs, and explicitly distinguish plant findings from inferences derived from yeast/animal systems. We also integrate recent advances on post-translational modifications (e.g., mitogen-activated protein kinase-dependent DCP1 phosphorylation) and RNA modifications (m^{6}A/ECT8) in selective mRNA targeting. Finally, we outline open questions regarding the spatial organization of decay, PB-stress granule crosstalk, and hormonal control mechanisms, and highlight methodological avenues to address them. Overall, plant PBs are presented as dynamic regulatory hubs that help tune post-transcriptional control in response to developmental and environmental cues, with their underlying mechanisms increasingly resolved by interdisciplinary strategies combining live-cell imaging, quantitative proteomics, and CRISPR-based genetics.

Key words: P-bodies, mRNA decay, translation repression, biomolecular condensates, stress responses, *Arabidopsis thaliana*

Matinahmadi A., Zayani Z., Majewska K., and Smoliński D.J. (2026). Plant P-bodies in post-transcriptional control: Composition, dynamics, and context-dependent roles. Plant Comm. **7**, 101787.

INTRODUCTION: UNDERSTANDING P-BODIES IN BIOLOGY

Processing bodies (P-bodies or PBs) are one class of cytoplasmic biomolecular condensate enriched by ribonucleoprotein (RNP) and act as highly dynamic, membrane-less organelles (MLOs). MLOs are cellular compartments that lack a delimiting lipid membrane and arise through diverse physicochemical mechanisms that locally concentrate proteins and RNAs. Biomolecular condensates are a subclass of MLO formed by multivalent interactions among proteins and nucleic acids. Such condensates can assemble via liquid–liquid phase separation (LLPS) but may also arise through other processes, such as scaffolded oligomerization or gelation (Alberti et al., 2019; Ismail et al., 2021; Zhang et al., 2023). Across eukaryotes, PBs concentrate decapping and 5′→3′ decay factors and can support translation repression; the relative contribution varies with cell type and condition (Teixeira et al., 2005; Vidya and Duchaine, 2022; Blake et al., 2024). Their formation and composition underscore the importance of compartmentalization in eukaryotic cells, helping maintain cellular homeostasis and responsiveness to changing conditions (Balagopal and Parker, 2009). PBs are found in a wide range of organisms, from simple yeast to complex mammals, highlighting a broadly conserved role in cellular biology (Nissan and Parker, 2008; Luo et al., 2018). PBs were initially discovered in yeast and found to be sites of mRNA degradation and storage (Sheth and Parker, 2003; Teixeira et al., 2005). Over time, this view has been refined by live-cell and single-molecule studies in mammalian cells, which show that PBs contribute to decay for a subset of transcripts but do not monopolize cytoplasmic mRNA turnover (Aizer et al., 2014; Tutucci et al., 2018; Wang et al., 2018). Subsequent research

 © 2026 The Author(s). Published by Elsevier Inc. on behalf of CAS Center for Excellence in Molecular Plant Sciences, Chinese Academy of Sciences, and Chinese Society for Plant Biology.
This is an open access article under the CC BY license (http://creativecommons.org/licenses/by/4.0/).

identified similar granules in mammalian cells, where they were shown to contain key mRNA-decay factors (Brengues et al., 2005; Liu et al., 2005; Teixeira et al., 2005). The identification of PBs in plants followed, with research demonstrating their presence in various plant species, including the model organism *Arabidopsis thaliana*. This discovery underscored the evolutionary conservation of these structures across eukaryotes (Weber et al., 2008; Xu and Chua, 2011). In addition to eukaryotes, bacterial ribonucleoprotein bodies exhibit conceptual similarities to eukaryotic PBs, raising the possibility that RNA–protein condensation as a regulatory strategy may have an ancient evolutionary origin (Ortiz-Rodríguez et al., 2025). In plants, PBs have adapted to fulfill specific roles in developmental programs and responses to environmental stresses (Maldonado-Bonilla, 2014; Liu et al., 2025). For instance, in *A. thaliana*, PBs participate in regulating the expression of stress-responsive genes, aiding adaptation to adverse conditions (Xiong et al., 2001; Weber et al., 2008; Xu and Chua, 2009). Plant PBs contain unique proteins, such as Decapping protein 5 (DCP5), absent in mammals, and are directly influenced by hormonal signaling pathways, including abscisic acid (ABA), during drought stress (Eulalio et al., 2007a; Xu and Chua, 2009; Covarrubias and Reyes, 2010; Maldonado-Bonilla, 2014). While certain aspects of PB composition may be plant specific, such as the presence of DCP5 in plants, direct functional comparisons with mammalian PB-associated processes, such as neuronal plasticity or immune responses, remain limited (Zeitelhofer et al., 2008a, 2008b; Riggs et al., 2020). Given the expanding body of research on PBs in plants, a comprehensive and organized synthesis is required to clarify their molecular components, assembly mechanisms, and roles in mRNA turnover, translation control, and stress adaptation. In this review, we summarize current knowledge of plant PBs, describe their biogenesis and composition, examine how they interface with other cytoplasmic regulatory compartments, and highlight their functional importance in mRNA decay and cellular homeostasis. We also discuss unresolved questions and future toolkits and directions that may deepen our understanding of PB-mediated RNA regulation in plants.

STRUCTURAL OVERVIEW OF PBs

This section focuses on the structural organization of plant PBs, starting from their core molecular composition, followed by the biophysical principles underlying their dynamic assembly as biomolecular condensates. We then discuss how post-translational modifications and signaling pathways modulate PB structure in response to environmental cues and finally place plant PBs in the broader context of cytoplasmic RNA granule networks.

Composition and architecture of PBs in plants

The composition of PBs is complex and heterogeneous, changes dynamically, and depends on the specific tissue, the organism, and the conditions within the cell. In plants, PBs are composed of a conserved core of mRNA-decapping and decay factors, including decapping enzymes (DCP), enhancers of decapping enzymes (EDC), exoribonuclease enzyme (XRN), DEAD-box helicases, and RNA-binding proteins (RBPs) together with plant-specific components that distinguish them from yeast and mammalian PBs (Table S1). While many structural elements are shared across eukaryotes, proteins such as DCP5 confer unique architectural and regulatory features to plant PBs. Of course, these components are also distributed around the cytoplasm and may be shared with some other cytoplasmic foci under certain conditions (Xu and Chua, 2009, 2011; Maldonado-Bonilla, 2014). The functional and physical PB protein associations in *A. thaliana* are visualized in Figure S1. Electron microscopy studies have revealed that PBs are spherical or irregularly shaped structures that can vary in size. The core of the PBs contains the main enzymatic machinery for mRNA decapping, surrounded by a shell of RBPs (Parker and Sheth, 2007). Studies in eukaryotic systems report that PBs range from ∼0.1 to 2 μm in diameter (Eulalio et al., 2007b; Teixeira and Parker, 2007; Aizer and Shav-Tal, 2008; Xu and Chua, 2009; Ayache et al., 2015; Rao and Parker, 2017). In mammalian cells, most cells contain approximately 3–9 distinct PBs, although both the number and size can vary substantially between cells. Smaller PBs are also likely to exist, as suggested by immunofluorescence studies. However, the functional significance of these size differences remains largely unclear. Variations in PB number and size are influenced by cellular conditions, including cell-cycle stage, proliferation status, and nutrient availability (Aizer and Shav-Tal, 2008). For example, in mouse oocytes, PBs appear larger in smaller oocytes, whereas in larger oocytes their size decreases, accompanied by an increased number of colocalizing foci observed in confocal sections (Flemr et al., 2010). Studies in yeast further indicate that PB size can be modulated by specific protein–protein interaction domains. Notably, PBs formed in Decapping protein 2 (DCP2) mutant strains are reproducibly smaller than those observed in Decapping protein 1 (DCP1) or exoribonuclease 1 (XRN1) mutant strains, highlighting the contribution of decapping components to PB architecture (Teixeira and Parker, 2007). In plants, direct quantitative measurements of PB size remain limited, and potential variability related to cell type or specific protein–protein interaction domains have not yet been systematically addressed.

Dynamic formation and dissolution of PBs as biomolecular condensates

At a conceptual level, plant PBs can be viewed as dynamic assemblies whose size, composition, and material properties allow cells to quickly adapt to changes in the environment or metabolic state. Rather than being static structures, they form and dissolve in response to changes in translation status, such as inhibition of translation initiation; stress signaling, such as heat stress or oxidative stress; and mRNA availability (Kedersha et al., 2005). The architecture of plant PBs is highly organized, allowing for the processing of mRNA molecules, and they are formed by the specific floating cytosolic proteins and non-translating mRNAs (Figure 1). The formation, maintenance, and function of PBs are tightly regulated through multiple mechanisms. These regulatory processes include post-translational modifications (PTMs) of the involved proteins, signaling pathways that influence their dynamics, and interactions with other cytoplasmic granules. This formation is driven by the dynamic condensation of RBPs and mRNAs, which can be triggered by various stress conditions or cell development (Teixeira et al., 2005; Balagopal and Parker, 2009; Xu and Chua, 2012). Before discussing how

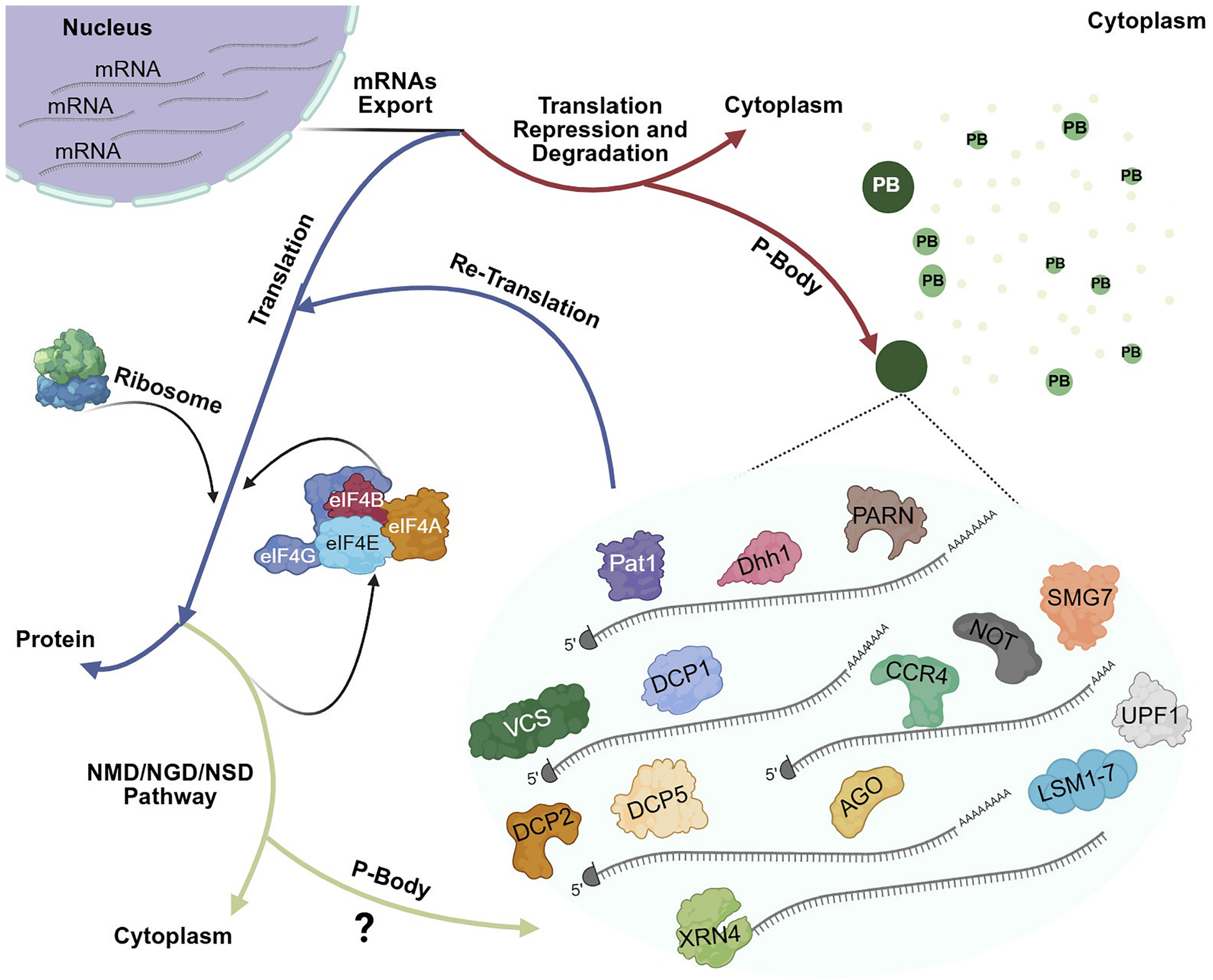

Figure 1. Overview of mRNA fate in the cytoplasm and the condensation of PB components.

Blue arrows indicate the translation process. Red arrows denote the translational repression and decay process. Green arrows refer to the co-translational decay process. P-bodies (PBs): light green spots in the top-right cytoplasm represent cytosolic proteins known as PB components, which have not yet assembled in PBs. Medium green foci in the top-right cytoplasm show the accumulation of these proteins, forming PBs of various sizes. Dark green foci in the top-right cytoplasm indicate bigger PBs. Exported mRNAs can undergo either translation or a repressed state and subsequently be decapped and degraded within the cytoplasm and PBs. In case of an event within the PBs, cytosolic proteins that facilitate PB assembly, together with mRNAs targeted for translation repression, form PBs of different sizes throughout the cytoplasm (magnified PB). On the other side, mRNAs intended for translation, along with eIFs and ribosomes, form polysomes and proceed with translation. mRNAs with translation defects are degraded via multiple pathways. Transcripts containing a Premature Termination Codon (PTC) are directed to nonsense-mediated decay (NMD). Meanwhile, mRNAs with stalled ribosomes in the coding region or those lacking a termination codon are targeted by no-go decay (NGD) and non-stop decay (NSD) pathways, respectively. These partially translated mRNAs may be transported to the PBs for degradation, although their transfer into plant PBs is still unclear. The re-translation of mRNAs from PBs allows the cell to rapidly adapt to changing conditions without transcribing new mRNAs, effectively "recycling" existing mRNAs in response to cellular needs. DCP1, mRNA-decapping enzyme subunit 1; DCP2, mRNA-decapping enzyme subunit 2; DCP5, Protein decapping 5; EDC4/VCS, Enhancer of mRNA decapping 4; ,LSM1–7, Sm-like proteins 1–7; XRN4, 5′→3′ exoribonuclease 4; PAT1, Protein associated with topoisomerase I; PARN, poly(A)-specific ribonuclease; Dhh1, DEAD-box helicases; SMG7, Nonsense-mediated mRNA-decay factor SMG7; CCR4-NOT, deadenylase complex; AGO, Argonaute protein; UPF1, Up-frameshift 1, eIF4A, Eukaryotic translation initiation factor 4A; eIF4B, Eukaryotic translation initiation factor 4B; eIF4G, Eukaryotic translation initiation factor 4G; eIF4E, Eukaryotic translation initiation factor 4E.

these principles apply to PBs, it is important to briefly outline the general biophysical basis of LLPS. Phase-separating RBPs often contain intrinsically disordered regions (IDRs), protein sequences that do not fold into a fixed three-dimensional structure, and do not expose defined secondary structures until they contribute to molecular interactions (Holehouse and Pappu, 2018; Zeke et al., 2022). Decapping factors are often modular and feature folded domains flanked or connected by low-complexity disordered regions. These disordered regions contribute to the assembly of decapping complexes and promote phase transitions that drive RNP granule formation. Phase transitions refer to the reversible conversion of dispersed proteins and RNAs into a condensed, liquid-like state in response to changes in cellular conditions (Jonas and Izaurralde, 2013). In Table S1, the proteins that include the IDRs have been marked, such as DCP5 (Wang et al., 2024), DCP2 (Maciej et al., 2022), and EDC4 (Jonas and Izaurralde, 2013). Some of these IDRs contain binding sites that promote multivalent

interactions or a large number of low-hydrophobic sequences that have the ability to drive proteins to undergo LLPS and thus condense into MLOs (Huang et al., 2024). The involvement of IDRs in phase transitions provides a biophysical angle to the characterization of proteins that harbor disordered regions (Van Der Lee et al., 2014). LLPS occurs when the multivalent interaction between RNA and proteins becomes energetically more favorable than interactions with the surrounding solvent. When these interactions collectively overcome the entropic cost of demixing, the molecules condense into a dense liquid phase, forming droplets. This process is highly sensitive to concentration, temperature, ionic strength, pH, and the charge patterning within IDRs of proteins. Electrostatic interactions, π–π contacts, and hydrophobic forces drive condensation, while salt and RNA can tune droplet viscosity and stability (Stroberg and Schnell, 2017). Consistent with these LLPS principles, PBs exhibit dynamic exchange of components with the surrounding cytoplasm and reversible assembly. In particular, PBs can range from more liquid-like to more arrested/gel-like states depending on the component and cellular context (Stroberg and Schnell, 2017). The extended and flexible structures of IDPs/IDRs enable them to engage in multiple transient interactions (Foressi et al., 2025). Many studies with deletion constructs have shown that the IDRs are sufficient and perhaps necessary for LLPS (Burke et al., 2015; Elbaum-Garfinkle et al., 2015; Lin et al., 2015; Molliex et al., 2015; Nott et al., 2015; Feric et al., 2016). Importantly, dysregulated phase transitions of RNP granules have been implicated in human neurodegenerative diseases such as Parkinson's disease and amyotrophic lateral sclerosis, underscoring the biological importance of maintaining proper material properties of condensates (Nedelsky and Taylor, 2019; Naskar et al., 2023).

The dissolution of PBs is equally important for cellular function, as it allows for the release and re-utilization of mRNA molecules when conditions improve. At the mechanistic level, since the PB assembly could be facilitated by weak multivalent protein–protein interactions among PB components bound to non-translating mRNAs, PB disassembly is thought to involve a combination of reduced multivalent interactions, remodeling of RNA–protein complexes, and changes in the physicochemical environment, such as ionic strength, pH, redox state, and temperature, that destabilize the condensed phase (Kim et al., 2021; Kearly et al., 2024). As these interaction networks collapse, stored mRNAs are released from the condensate, rendering them accessible to the translational machinery or alternative RNA decay pathways. This process often involves the action of ATP-dependent mechanisms (e.g., helicases, chaperones) that disassemble the protein and RNA aggregates within PBs. ATP-dependent RNA helicases can actively remodel messenger ribonucleoproteins (mRNPs) by unwinding RNA structures and displacing RBPs, thereby weakening the interaction network that maintains the PBs condensate (Buchan, 2024). Moreover, the depletion of LSM1, RCK/p54, eIF4E-T, and proteins that are involved in microRNA (miRNA) processing, such as Drosha and its binding partner DGCR8, results in the loss of mammalian PBs (Eulalio et al., 2007a). Regulated dissolution of PBs can facilitate re-entry of sequestered transcripts into translation. This controlled disassembly provides a rapid and reversible mechanism for translational reprogramming, enabling cells to efficiently transition from stress-induced repression to active protein synthesis upon recovery (Franks and Lykke-Andersen, 2008). However, direct experimental evidence dissecting the material properties and phase behavior of individual plant PB components remains limited.

Signaling-driven post-translational modification in PB assembly and composition

PTMs and signaling pathways operate as a tightly coupled regulatory axis that governs PBs assembly, disassembly, dynamics, and functional specialization. These modifications directly alter protein–protein and protein–RNA interactions, thereby modulating LLPS, PB composition, and the balance between mRNA storage, translational repression, and decay (Hofweber and Dormann, 2019; Sternburg et al., 2022). PB dynamics respond rapidly to diverse environmental and metabolic inputs, including heat shock, oxidative stress, nutrient deprivation, and hormonal signaling (Rzeczkowski et al., 2011). In eukaryotic systems, major kinase-driven pathways such as the mammalian target of rapamycin (mTOR), mitogen-activated protein kinase (MAPK), and c-Jun N-terminal kinase cascades have been implicated in PB regulation. Under nutrient-rich conditions, active mTOR signaling suppresses PB formation and favors translation, whereas stress-induced inhibition of mTOR promotes PB enlargement, partly through effects on the stability of scaffold protein RCD-8 (EDC4) (Gudkova et al., 2011). Likewise, perturbation of TAK1–Jun N-terminal kinase signaling alters PB number, size, and the subcellular localization of core components, including DCP1, XRN1, and EDC4 (Rzeczkowski et al., 2011). In plants, direct evidence for mTOR-mediated PB control remains limited and largely indirect; however, stress-responsive MAPK pathways are well established as key regulators of PB behavior. Stress signals such as drought or salinity can activate MAPKs, which then influence PB composition by phosphorylating key proteins involved in mRNA decay and translation repression (Xu and Chua, 2012; García et al., 2019; Huang et al., 2024).

The principal molecular output of these signaling cascades is the post-translational modification of PB proteins, particularly those enriched in IDRs. PTMs can alter IDR charge, hydrophobicity, size, and conformational flexibility through the addition of chemical groups (e.g., phosphoryl, methyl, acyl, glycosyl, alkyl) or subtler chemical changes such as ubiquitination, oxidation, deimidation, and deamidation (Bah and Forman-Kay, 2016; Li et al., 2017). As a result, PTMs function as molecular switches that fine-tune PB material properties and selectively reshape their protein and RNA composition in response to cellular signals (Hofweber and Dormann, 2019; Kim et al., 2021).

Broader research in other eukaryotes provides a roadmap for what might be discovered in plants. In yeast and mammalian cells, phosphorylation of various PB components, such as DCP2, PAT1, and LSM proteins, is known to regulate their activity and localization (Eulalio et al., 2007a; Kedersha and Anderson, 2009; Yoon et al., 2010; Xu and Chua, 2012; Aizer et al., 2013; Standart and Weil, 2018). In plants, the best-characterized example is the phosphorylation of UPF1 during nonsense-mediated decay (NMD). When SMG7 is available, the SMG7-UPF1

Pathway	Alias	Key features	Main enzymes/factors	Relation to PBs	References
Deadenylation and decapping-dependent decay	–	● shortening of the poly(A) tail mainly results in the removal of the 5′ cap structure<br>● 5′→3′ degradation by exonuclease	5′→3′: DCP1, DCP2, DCP5, EDC4, XRN4<br>3′→5′: ,LSM1–7, Pat1, Dhh1, PARN, NOT, CCR4	because decapping factors are enriched in PBs, many steps can occur there; however, parts of the pathway also proceed diffusely in the cytoplasm	(Chen et al., 2011; Ling et al., 2011; Passmore and Coller, 2022)
5′→3′ co-translational decay	Xrn4-mediated co-translational decay	● can occur in a deadenylation-independent manner and probably does not require prior removal of the poly(A) tail<br>● 5′→3′ mRNA degradation occurs alongside the ribosome's codon-by-codon movement during translation<br>● XRN4 likely executes co-translational decay once translation initiation is inhibited, either before or as a result of 5′-cap removal<br>● Occurs where ribosomes pause or stack.<br>● May be tied to how quickly ribosomes move along the mRNA, targeting mRNAs that slow down during elongation or termination.	5′→3′: XRN4, DCP1, DCP2, DCP5, EDC4	if cap removal triggers this decay, it may involve PBs, although the exact role of PBs in co-translational decay remains unclear	(Merret et al., 2015; Hou et al., 2016; Yu et al., 2016; Crisp et al., 2017; Chantarachot and Bailey-Serres, 2018; Han et al., 2023; Carpentier et al., 2024)
3′→5′ co-translational decay	● decapping-independent decay<br>● exosome-Mediated 3′→5′ decay	● noncanonical pathway of mRNA degradation that bypasses the removal of the 5′ cap<br>● it can take place parallel to or outside the canonical 5′→3′ pathway<br>● after deadenylation, the 3′ ends of mRNAs are degraded by the exosome	deadenylation: CCR4, NOT, PARN<br>SKI complex: SKI8, SKI3, SKI2<br>exosome: RRP41, RRP42, RRP43, RRP45, RRP46, RRP41L/MTR3, RRP4, RRP40, CSL4	SKI proteins are not considered a general component of PBs, but their presence in PBs relies on association with some other PB components<br>due to the absence of decapping factors in this pathway, it is more likely to take place throughout the cytoplasm rather than being targeted to PBs	(Chekanova et al., 2002, 2007; Zhao and Kunst, 2016; Chantarachot and Bailey-Serres, 2018; Weick and Lima, 2021; Li et al., 2023)

Table 1. mRNA degradation pathways and their interplay with PBs in plants.

(Continued on next page)

Pathway	Alias	Key features	Main enzymes/factors	Relation to PBs	References
Endonucleolytic cleavage	–	● internal cleavage before decapping and deadenylation<br>● plays a crucial role in RNA-mediated gene silencing (RNAi)<br>● mRNAs are divided into two fragments, one containing a new 5′ end and the other containing a new 3′ end	endonucleases:<br>in RNA-mediated gene silencing: Argonaute (AGO)<br>5′→3′: XRN4<br>3′→5′: exosome, SKI complex	in plants, the occurrence of mRNA cleavage within PBs remains unknown	(Chan and Slack, 2006; Xu and Chua, 2009; Mérai et al., 2013; Szádeczky-Kardoss et al., 2018)
Nonsense-mediated mRNA decay (NMD)	faulty transcript decay	● translation-dependent process that removes mRNAs with PTCs<br>● these faulty mRNAs are degraded through decapping, bypassing the usual requirement for poly(A) tail shortening<br>● unlike the animals and yeast, NMD in plants does not initiate via endonucleolytic cleavage near the PTC<br>● in plants, NMD has two main phases: conserved (early step) and non-conserved (late steps)<br>● it is hypothesized that the early steps of NMD are highly conserved, whereas the late steps are unique to plants<br>● the early step begins with the processing of pre-mRNAs. The mature mRNAs are exported from the nucleus to the cytoplasm	early step: nuclear pore complex (NPC), TREX, Upf3, exon junction complex (EJC), PYM factors (the protein that interacts with the exon junction)<br>late step (absence of SMG7): UPF1, XRN4, PABP, eRF1<br>late step (presence of SMG7): UPF1, SMG7, XRN4	the role of PBs in plant NMD has not been verified. SMG7 in plants can lead to the relocation of Upf1 into the PBs, which means that the SMG7-UPF1 pathway, in late steps, can be located inside the PBs in comparison with the UPF1-XRN4 pathway	(Coller and Parker, 2004; Sheth and Parker, 2006; Brogna and Wen, 2009; Jeong et al., 2011; Mérai et al., 2013; Dai et al., 2016; Zhang and Guo, 2017)

Table 1. ***Continued***

(Continued on next page)

Pathway	Alias	Key features	Main enzymes/factors	Relation to PBs	References
		• late steps are classified into two main NMD pathways. UPF1-XRN4 pathway (absence of SMG7), XRN4 is triggered to initiate 5′→3′ mRNA decay. Alternatively, the absence of SMG7 can activate PABP (PolyA binding protein) and eRF1, leading to 3′→5′ mRNA degradation<br>SMG7-UPF1 pathway (presence of SMG7), in which phosphorylated UPF1 activates this pathway			
No-go decay (NGD) and non-stop decay (NSD)	• translation-dependent surveillance decay<br>• mRNA quality-control mechanisms	• NSD degrades two types of stop-codon-free mRNAs: non-stop and stop-codon-less mRNAs<br>• NGD identifies and eliminates mRNAs with structural impediments that block translation elongation<br>• both pathways play a role in resolving stalled ribosomes, facilitating their dissociation and recycling from defective transcripts<br>• in both pathways, the mRNAs are degraded when ribosomes stall upstream of stop codons<br>• NGD leads to endonucleolytic cleavage	NSD: SKI complex, SKI7<br>NGD: 5′ fragments are degraded in a Pelota-HBS1- and SKI2-dependent manner<br>3′ fragments are degraded by XRN4	in plants, the occurrence of NGD and NSD within PBs remains unknown	(Garneau et al., 2007; Jackson et al., 2012; Zhang et al., 2015; Chantarachot and Bailey-Serres, 2018; Szádeczky-Kardoss et al., 2018; Kong et al., 2021)

Table 1. *Continued*

pathway will be activated by phosphorylated UPF1 (Table 1), resulting in the relocalization of PBs and XRN4-mediated 5′→3′ decay of the mRNA, linking signaling-dependent phosphorylation to PB-associated mRNA turnover (Dai et al., 2016). Another plant example of signaling-driven PTM involves MAPK-dependent phosphorylation of the decapping factor DCP1 during immune responses to bacterial pathogens. This phosphorylation event promotes PB disassembly by stimulating mRNA decapping and 5′→3′ exonucleolytic decay, reflecting dynamic remodeling of PB composition and function rather than simple inhibition or activation (Yu et al., 2019; He et al., 2024). Such modification is likely to influence DCP1 interactions with decapping partners, including DCP2, DCP5, and VCS, thereby regulating the recruitment or release of decay enzymes and translational repressors. Consistent with this model, VCS itself is a phosphoprotein, supporting the view that coordinated phosphorylation of multiple PB components modulates PB composition and functional output under stress conditions (Huang et al., 2024). Beyond phosphorylation, other PTMs are emerging as regulators of PB dynamics. In yeast and mammalian systems, arginine methylation of the N-terminal LSM4 RGG domain enhances PB assembly by modifying hydrophobic and hydrogen-bonding interactions (Owen and Shewmaker, 2019), while K63-linked ubiquitination of protein HAX1 by E3 ligase TRIM23 promotes PB condensation under energy stress by stabilizing interactions among PB-associated factors, such as DDX6 (Kedia et al., 2022; Zhan et al., 2024). Although direct evidence for comparable ubiquitination- or methylation-driven mechanisms in plants is currently lacking, the conservation of PB architecture and protein quality-control pathways suggests that similar PTM-based regulatory strategies are likely to operate in plant cells.

Hormonal signaling provides an additional layer of integration with PB regulation. Plants are known to produce ethylene in response to numerous environmental stress conditions. Ethylene is a gaseous plant-growth regulator that controls a multitude of developmental and stress responses (Olmedo et al., 2006). Ethylene signaling activates the endoplasmic reticulum, localized protein Ethylene Insensitive 2 (EIN2), which facilitates the targeting of specific transcripts, such as EIN3-Binding F-BOX 1 (EBF1) mRNA, to cytoplasmic PBs (Li et al., 2015; Merchante et al., 2015; Riyazuddin et al., 2020; Kim et al., 2021). While this mechanism does not directly involve PTMs of PB proteins, it illustrates how signaling pathways converge on PBs by modulating their RNA cargo, complementing kinase-driven PTM-mediated control of PB assembly and composition. Collectively, these findings underscore that signaling pathways regulate plant PBs primarily through PTM-dependent remodeling of their molecular constituents, thereby linking upstream signal perception to downstream post-transcriptional decisions.

Interplay with other cytoplasmic granules: Stress granules and beyond

Although PBs serve as major biomolecular condensates for mRNA turnover, they do not exist in isolation. Other RNA granules, such as stress granules (SGs), also play significant roles in regulating mRNA metabolism, often collaborating with PBs to manage mRNA fate. While PBs are constitutively present in the cell, SG formation is a stress-induced event (Parker and Sheth, 2007; Chantarachot et al., 2020; Kearly et al., 2024). SGs form in response to cellular stress and primarily sequester mRNAs that are stalled in translation initiation. These mRNAs are protected from degradation and can later be re-engaged in translation once the stress has subsided. Although SGs and PBs have distinct roles, they often coexist within the same cellular regions, and their components can dynamically exchange in different organisms (Kearly et al., 2024). Notably, studies in yeast have shown that pre-existing PBs enhance the assembly of SGs, and mutations that strongly impair PB formation significantly inhibit SG formation, indicating a functional dependency of SG assembly on PBs (Brengues and Parker, 2007; Hoyle et al., 2007; Buchan et al., 2008). Consistent with this model, mRNAs exiting translation often transit through PBs before being recruited into SGs, supporting a directional flow from PBs to SGs and highlighting PBs as integrative hubs for stress-induced mRNA regulation (Buchan et al., 2008). While most evidence for PB-dependent SG assembly comes from yeast and mammalian systems, similar interactions are likely to occur in plants, although direct experimental evidence is still lacking. This is supported by the conservation of core PB components and their observed co-localization with SGs. It is worth noting that, beyond cytoplasmic SGs, plastids (including chloroplasts) also assemble stress-induced RNA granules, adding an additional layer of post-transcriptional regulation in plant cells. Chloroplast SGs (cpSGs) were identified in *Chlamydomonas reinhardtii*, where high-light stress triggers the formation of distinct RNA–protein condensates within the chloroplast stroma (Uniacke and Zerges, 2008). Similar to cytoplasmic SGs, cpSGs recruit translationally repressed mRNAs and RBPs; however, they also display plastid-specific features, such as their proximal localization to thylakoid membranes and their involvement in regulating chloroplast translation. More recent proteomic and imaging analyses in *A. thaliana* have demonstrated that heat stress rapidly induces cpSG assembly, which sequesters selected chloroplast-encoded mRNAs, metabolic enzymes, RBPs, ATPases, chaperones, and translation elongation factors (Chodasiewicz et al., 2020). Importantly, because cpSGs form within plastids, whereas PBs are cytoplasmic condensates, no direct functional interactions between these two granule types have been observed to date.

In *A. thaliana*, during heat shock at 35°C, cytoplasmic SGs begin to form around 70 s after temperature elevation, appearing both adjacent to PBs and independently of them. Initially, only about half of the PBs are associated with SGs, and some remain unassociated even after 250 s. SGs rapidly increase in number and frequently fuse, as well as with PBs, although the overlap between the two structures is not complete (Hamada et al., 2018). Once SGs associate with PBs, they remain stably attached, but their sizes are not correlated. Over prolonged heat exposure (60–120 min), SGs continue to fuse, leading to fewer but larger SGs. Despite this, PBs largely remain separate and are not incorporated into the large SGs, suggesting that SG–SG interactions are stronger than SG–PB interactions (Hamada et al., 2018). Overall, SG formation involves two distinct steps, generation and fusion, and occurs independently of PBs, although transient associations between the two structures do occur (Hamada et al., 2018). One major point of interaction between SGs and PBs in yeast and

mammals is through their local components, proteins such as DDX6 (Dhh1) and Pat1, which are central to PB function, are also found in SGs, indicating a shared pool of mRNA-binding proteins (Hilliker, 2012; Guzikowski et al., 2019). Under stress conditions in plants, the tandem zinc-finger proteins TZF1, TZF4, TZF5, and TZF6 interact physically in both SGs and PBs (Pomeranz et al., 2010a; Bogamuwa and Jang, 2016). In addition, mRNAs can shuttle between SGs and PBs, determining whether the mRNA is stored for future translation or degraded. This cooperative mRNA regulation exemplifies a broader cellular strategy in plants (Weber et al., 2008; Chantarachot and Bailey-Serres, 2018; Youn et al., 2019; Kearly et al., 2024). Such interactions highlight the versatility of plant PBs as integrative centers linking translational arrest to targeted degradation. This dynamic interplay is particularly critical in plants for adapting to abiotic stresses such as heat and drought, which represents a key functional distinction from mammalian systems, where such crosstalk is often associated with immune responses or pathological states. Collectively, these structural features highlight that plant PBs are not passive aggregates but highly organized, signal-responsive condensates whose architecture underpins their functional versatility in post-transcriptional regulation.

FUNCTIONAL OVERVIEW OF PBs

Unless stated otherwise, functional examples below refer to *A. thaliana*; non-*Arabidopsis* cases (e.g., *Oryza sativa*) are explicitly labeled.

A synthesis of key plant PB studies across formation, mRNA regulation, stress responses, and development is provided in Table 2, and the main findings from these papers are integrated into the corresponding subsections of this section (from sections role of plant PBs in mRNA degradation and turnover to developmental roles of PBs across plant life stages).

Role of plant PBs in mRNA degradation and turnover

While PBs concentrate decapping and exonucleolytic factors and can facilitate decay or storage of non-translating mRNAs, decapping and decay also occur outside large, microscopically visible PBs, including in smaller, diffraction-limited assemblies and the bulk cytosol (Sheth and Parker, 2003; Coller and Parker, 2004; Wang et al., 2018). Early biochemical and imaging studies already suggested that decapping can proceed in the cytoplasm without the need for visible cytoplasmic compartmentalization (Horvathova et al., 2017; Schütz et al., 2017). Live-cell and single-molecule imaging in animal systems further indicate that a substantial fraction of mRNA decapping and degradation proceeds in a diffuse cytoplasmic pool rather than within morphologically defined PBs (Tutucci et al., 2018; Wang et al., 2018). Therefore, PBs are neither the sole nor universally essential sites for these processes, and their precise contribution remains a subject of investigation. This has led to a key debate around whether PBs are sites of active decay or primarily storage hubs for repressed mRNAs that are later targeted for degradation elsewhere. Quantitative tracking in living cells shows that only a subset of transcripts that visit PBs are degraded there, whereas many decay events occur in the surrounding cytoplasm, consistent with a dual storage-and-decay role (Aizer et al., 2014; Wang et al., 2018). While some studies therefore suggest that PBs primarily concentrate decay enzymes without exclusively restricting their activity to these sites (Franks and Lykke-Andersen, 2008; Decker and Parker, 2012), a compelling perspective is that the condensation of decay factors within PBs biases selection and kinetics for specific mRNA subsets, thereby improving the coordination and efficiency of turnover (Vidya and Duchaine, 2022). For instance, using rapid inducible decay of RNA in osteosarcoma cell lines and mouse embryonic fibroblasts, the RNA decay dynamics in cells were tracked, and it was found that mRNAs degrade more rapidly in PBs than in the cytoplasm. Upon induction, target mRNAs were quickly localized to PBs, where they underwent fast degradation, compared to the slower decay observed in the cytoplasm. Moreover, knocking down key PB proteins and RNA degradation enzymes confirmed that PBs actively contribute to RNA decay, which was the first study to measure RNA degradation kinetics in different cellular compartments, highlighting the role of PBs as specialized sites for rapid RNA degradation (Blake et al., 2024). These rapid inducible decay of RNA-based measurements are consistent with earlier imaging studies showing that PB association accompanies decay for a subset of targets but is not obligatory for all decay events (Aizer et al., 2014; Tutucci et al., 2018; Wang et al., 2018). While these kinetics provide strong evidence in animal cells, direct, compartment-resolved decay rates in plants remain to be established. However, a body of indirect evidence in plants suggests a critical functional role for PB-associated machinery. In *A. thaliana*, mutants defective in core mRNA-decapping factors, such as DCP1, DCP2, and DCP5, which are enriched in PBs but also function in the cytosol, show accumulation of specific capped mRNAs accompanied by severe developmental phenotypes, including seedling lethality (Iwasaki et al., 2007; Xu and Chua, 2011). Furthermore, PB assembly, which involves components such as DCP5, has been implicated in modulating the efficiency of decapping for selected transcripts in plants (Xu and Chua, 2009), although decapping can also proceed outside large, microscopically visible PBs. The relative contribution of PB-enriched steps versus cytoplasmic decay remains context dependent and is influenced by developmental stage and stress conditions. While elegant single-molecule imaging studies in *Drosophila* have demonstrated a clear role for PBs in facilitating 5′→3′ degradation (Forbes Beadle et al., 2023), direct evidence for active enzymatic decay occurring within plant PBs remains elusive.

mRNA degradation is not exclusively triggered by specific cellular conditions; it is critical for sustaining proper gene expression, cellular homeostasis, and quality control. Even under non-stressed conditions, mRNA turnover ensures that only properly structured transcripts are retained, while faulty transcripts, such as from splicing errors, are rapidly degraded (Coller and Parker, 2004; Eulalio et al., 2007a; Chen and Shyu, 2011; Roy and Rajyaguru, 2018). Plants, like other eukaryotes, utilize translation-dependent RNA quality-control pathways to preserve translational accuracy (Zhang and Guo, 2017). The decapping and deadenylation-dependent pathway is a major contributor to general mRNA turnover in plants (Sorenson et al., 2018). This process involves a series of coordinated steps where core PB components are implicated. Typically, a protein like DCP5 first associates with target mRNAs,

Category	Study description	Species	Plant-specific feature	References
**Formation and composition**	disruption of the decapping complex (VCS, DCP2) impedes PB formation.	*A. thaliana*	highlights the essentiality of the core decapping machinery for PB integrity	(Goeres et al., 2007)
	the *Arabidopsis* decapping complex (DCP1, DCP2, VCS) assembles *in vitro* and *in vivo*, localizing to PBs	*A. thaliana*	confirms the conserved nature of the decapping complex assembly	(Xu et al., 2006)
	DCP5 is essential for PB formation and the recruitment of mRNPs into condensates	*A. thaliana*	DCP5 is a key plant-specific scaffold protein absent in mammals	(Xu and Chua, 2009)
	AtTZF proteins co-localize with PB and SG markers (DCP2, XRN4, PABP), suggesting a role in RNA turnover	*A. thaliana*	tandem zinc-finger proteins are important stress-related RNA binders in plants	(Pomeranz et al., 2010b)
**mRNA regulation**	PBs facilitate mRNA degradation and modulate gene expression	*A. thaliana*	confirms a conserved, fundamental role in post-transcriptional control	(Bhullar et al., 2017)
	mutants in decapping components lead to the accumulation of capped mRNAs	*A. thaliana*	provides direct genetic evidence for the role of PBs in mRNA decay	(Goeres et al., 2007)
	PBs suppress the translation of a large set of mRNAs (∼20%) in dark-grown seedlings	*A. thaliana*	demonstrates a major regulatory role during a key developmental transition (skotomorphogenesis)	(Jang et al., 2019)
	the LSM1–7 complex promotes the degradation of stress-responsive transcripts within PBs	*A. thaliana*	links a core PB complex to selective turnover of stress-related mRNAs	(Perea-Resa et al., 2016)
**Stress response**	PBs increase in number and size in response to various stresses	*A. thaliana*	a conserved hallmark of the cellular stress response	(Bhullar et al., 2017)
	XRN4 and LARP1 co-localize to PBs during heat stress to regulate heat-sensitive mRNAs	*A. thaliana*	highlights a specific mechanism for thermal stress adaptation	(Merret et al., 2013)
	dehydration stress triggers MPK6-mediated phosphorylation of DCP1, which modulates PB assembly.	*A. thaliana*	direct link between MAPK signaling and PB dynamics in response to abiotic stress	(Xu and Chua, 2012)
	VCS and SRK2G/SnRK2.1 associate in PBs under osmotic stress	*A. thaliana*	integrates PBs with ABA-independent osmotic stress signaling (SnRK2)	(Soma et al., 2017, 2020)
	Tudor staphylococcal nuclease is a stress-induced PB component required for efficient mRNA decapping	*A. thaliana*	identifies a stress-inducible factor that enhances PB function	(Gutierrez-Beltran et al. 2015)

Table 2. Key findings in plant PB research from 2004–2025 with plant-specific features highlighted.

(Continued on next page)

Category	Study description	Species	Plant-specific feature	References
	OsTZF1, a CCCH-tandem zinc-finger protein, localizes to cytoplasmic foci under stress conditions	*O. sativa*	shows conservation of stress-related mechanisms in a major crop species	(Jan et al., 2013)
	identification of a wheat rust fungal effector (PST02549) that localizes to plant PBs and interacts with the PB protein EDC4, suggesting manipulation of host mRNA metabolism and immune responses	Wheat (*Triticum aestivum*) and *N. benthamiana*	interaction with plant EDC4 and enlargement of PBs, linking PBs to plant immunity and host–pathogen interactions	(Petre et al., 2016)
	PB-localized RNA helicase BnRH6 modulates salt stress responses by regulating expression of stress- and ABA-responsive genes	*B. napus* and *A. thaliana*	PB localization of a plant DEAD-box RNA helicase regulating salt stress tolerance and ABA-responsive pathways	(Zhang et al., 2022)
	HuTZF3 localizes to PBs and SGs and contributes to salt and heat tolerance	*H. polyrhizus* (Pitaya)	PB localization improves salt and heat tolerance	(Xu et al., 2023)
**Motility and localization**	PB movement in the cytoplasm is actin dependent and mediated by myosin XI	*A. thaliana*	reveals the mechanism of PB transport, which is crucial for their function in large plant cells	(Steffens et al., 2014)
	PBs show actin-based motility and are partitioned into daughter cells during protoplast reprogramming and cell division.	*N. tabacum* (mesophyll protoplasts)	actin–myosin XI–dependent PB movement and equal partitioning during plant cell division; contrast with microtubule-based motility in animal cells	(Bhullar et al., 2017)
**Developmental roles**	PBs regulate meiotic exit by mediating the translational repression of key transcripts.	*A. thaliana*	a critical role in the plant reproductive cycle	(Cairo et al., 2022)
	DCP5 is required for repressing the translation of SSP mRNAs during germination.	*A. thaliana*	essential for clearing maternal mRNAs during the seed-to-seedling transition	(Xu and Chua, 2009)
	core decapping components (DCP1, DCP2, VCS) are essential for postembryonic development	*A. thaliana*	confirms the fundamental importance of PB machinery for plant viability	(Xu et al., 2006; Goeres et al., 2007)

Table 2. ***Continued***

repressing their translation and facilitating the recruitment of the decapping complex, including VCS (EDC4) and DCP1 (Xu and Chua, 2009). DCP1 then acts as a scaffold to recruit the catalytic enzyme DCP2, which removes the 5′ cap, a regulated, irreversible step. Once uncapped, the mRNA is degraded by the 5′→3′ exonuclease XRN4 (Xu and Chua, 2011). This cascade is further modulated by the ‚LSM1–7 complex, which binds to the 3′ end of oligoadenylated mRNAs, and proteins such as Pat1 and DDX6 (Dhh1), which contribute to translational repression and recruit the decapping machinery (Tharun and Parker, 2001; Perea-Resa et al., 2012; Sharif et al., 2013; Roux et al., 2015). Although the key proteins of this pathway are enriched in PBs, the decay process itself is not confined to these structures (Figure 2, green pathway; Table 1).

Crucially, the targeting of mRNAs to this decay pathway is not random but selective, with specific transcripts being marked for

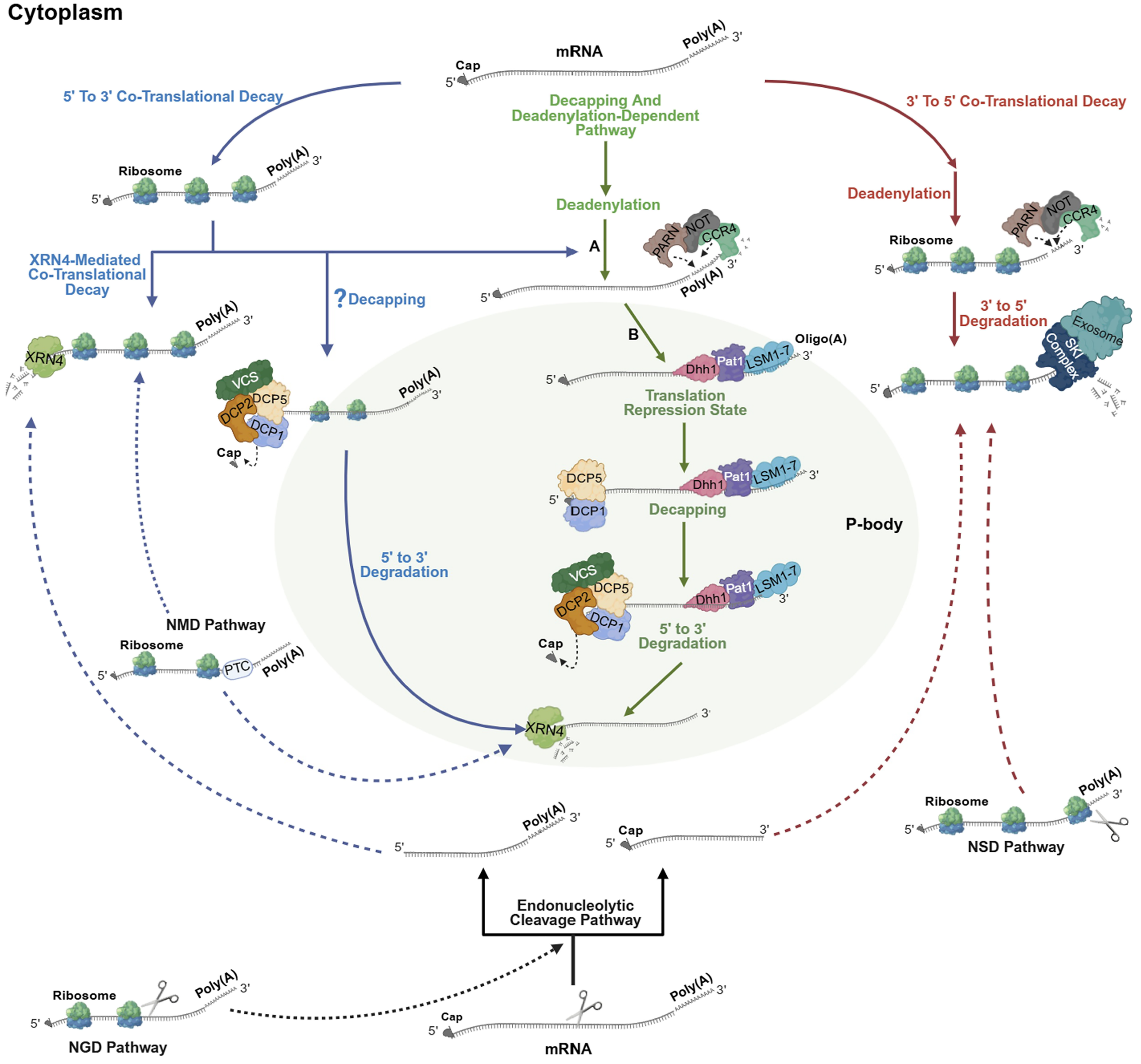

Figure 2. A model of mRNA degradation pathways and their proposed association with PBs in plants.
This schematic represents a model integrating multiple mRNA-decay pathways (Dai et al., 2016; Zhang and Guo, 2017; Chantarachot and Bailey-Serres, 2018). It should be emphasized that, while the core protein components are localized to PBs, not all enzymatic steps depicted within the PB boundary have been directly and unequivocally demonstrated to occur inside plant PBs. Some steps, such as deadenylation, primarily occur in the cytoplasm before mRNA localization to PBs, while the precise location of others remains an active area of research. 5′→3′ co-translational decay (blue pathway): this pathway can occur with or without prior deadenylation. The degradation of mRNA by the 5′→3′ exonuclease XRN4 happens concurrently with translation, often when ribosomes stall. This process may involve cap removal, which implicates decapping factors within the cytoplasm, although a direct role for PBs in this specific pathway is still unclear in plants. The NMD pathway in plants is also considered a 5′→3′ decay process.Decapping and deadenylation-dependent pathway (green pathway): this is a major mRNA degradation route. It begins in the cytoplasm with the shortening of the poly(A) tail by deadenylases such as the PARN and CCR4-NOT complexes (A). The resulting oligoadenylated mRNA is then recognized by the ,LSM1–7-Pat1-Dhh1 complex and localizes to PBs (B). There, the mRNA is held in a translationally repressed state, and the decapping complex (DCP1/DCP2/DCP5/VCS) removes the 5′ cap. Following decapping, the exonuclease XRN4 degrades the mRNA from the 5′ end.3′→5″ co-translational decay (red pathway): this pathway bypasses the need for decapping. After deadenylation in the cytoplasm, the mRNA is degraded from the 3′ end by the exosome, a process facilitated by the SKI complex. While SKI complex components can associate with PBs, the 3′→5′ decay is generally considered a cytoplasmic process. The NSD pathway is an example of 3′→5′ decay. Endonucleolytic cleavage pathway (black pathway): this pathway is initiated by an internal cleavage of the mRNA, generating two fragments that are subsequently degraded by XRN4 (5′→3′) and the exosome (3′→5′). This is a key mechanism in RNA-mediated gene silencing (RNAi) and NGD. While plant NMD is not initiated by endonucleolytic cleavage (unlike in some other eukaryotes), the subsequent degradation of cleaved fragments involves general decay machinery. Note: this is a

(legend continued on next page)

rapid turnover in response to particular signals (Zheng et al., 2008; Zhou et al., 2024). Recent research has revealed further layers of complexity, flexibility, and heterogeneity, showing that the predominant mRNA-decay pathway varies between different plant organs. In *A. thaliana* shoots, most mRNAs undergo co-translational 5′→3′ decay by XRN4 while still associated with ribosomes. In roots, however, a compensatory mechanism exists where the loss of this co-translational branch triggers an accelerated ribosome-free 5′→3′ decay. Thus, although both organs use the same core enzymes, shoots rely predominantly on co-translational turnover, whereas roots balance and compensate between the two pathways (Carpentier et al., 2024).

Role of plant PBs in selective mRNA translation and storage

The dual functionality of PBs allows cells to dynamically regulate gene expression and adjust rapidly to fluctuating conditions, sequestering mRNAs for either storage or eventual degradation in response to cellular conditions (Wang et al., 2018). For instance, when stress subsides, sequestered mRNAs can be released from PBs and re-enter the active translation pool, allowing for a swift recovery without the need for *de novo* transcription (Parker and Sheth, 2007). PBs can buffer the cellular translation pool by temporarily sequestering non-translating mRNAs. Sequestering non-translating mRNAs and decay machinery into PBs may offer several advantages. First, it partitions the decapping machinery from actively translating mRNAs, preventing premature degradation. This compartmentalization likely provides an additional layer of regulatory control (Coller and Parker, 2004). Second, PBs may serve as a cellular buffering system to maintain the balance between translational capacity and the pool of available mRNAs. By sequestering non-translating mRNAs, the cell can ensure more efficient translation of the remaining transcripts (Figure 3). This buffering system may have evolved additional roles in regulating mRNA fate (Coller and Parker, 2004; 167). Third, it has been suggested that translational repression occurs in two phases: an initial slowdown followed by a second phase where repression is fully established, which could correspond to sequestration within PBs (Coller and Parker, 2004).

Evidence in plants points to selective effects on stress-related transcripts and key developmental programs, such as seed germination. This function is particularly evident under stress conditions such as heat shock or nutrient limitation, where global translation rates decline. Untranslated mRNAs accumulate in PBs, enabling the cell to conserve energy while retaining key transcripts for potential later use (Passmore and Coller, 2022). PBs might function as an "isolator" to segregate specific groups of mRNAs from the general cytoplasm, marking them for degradation in response to cellular needs (Wang et al., 2018). The absence of the 3′→5′ decay machinery from PBs (Table 1; Figure 2) may also help direct specific mRNAs toward the 5′→3′ decay pathway (Hubstenberger et al., 2017; Youn et al., 2018; Lee et al., 2020). The mechanisms by which specific mRNAs are selected for distinct degradation pathways are actively being investigated. In eukaryotes, RNA modifications are emerging as a key selection mechanism. For example, m6A in the coding sequence (CDS) triggers translation-dependent mRNA decay via CDS-m6A decay, and refers to the potential PB involvement, and recruitment of YTHDF2 for accelerated degradation (Fu et al., 2014; Lee et al., 2020; Zhou et al., 2024; Murakami et al., 2025). Recent findings in plants provide a compelling mechanism for this selectivity. In *A. thaliana*, evolutionarily conserved C-terminal region 8 (ECT8) was identified as an m6A reader protein and showed that its m6A-binding capability is required for salt stress responses. ECT8 accelerates the degradation of its target transcripts through direct interaction with the DCP5 within PBs (Cai et al., 2024). This provides a plant-specific route for the selective turnover of modified transcripts, linking environmental stress to post-transcriptional control within these condensates. Moreover, in *A. thaliana*, the reduction of PBs in light-grown *RH6*, *RH8*, and *RH12* mutant seedlings results in elevated accumulation and translation of stress-related mRNAs under non-stress conditions, indicating that PBs help suppress these specific transcripts under normal conditions (Chantarachot et al., 2020).

The sequestered mRNAs are held in a translationally repressed state through the action of multiple factors. In *A. thaliana* and *O. sativa*, the CCR4-CAF1 deadenylase complex plays a role in this process by shortening poly(A) tails, a common precursor to translational repression (Walley et al., 2010; Chou et al., 2014). Within PBs, other repressors such as Pat1, DDX6/Dhh1, and components of the ,LSM1–7 complex are thought to prevent ribosome attachment by binding mRNAs and unwinding secondary structures. While deadenylation is often a prerequisite for repression and subsequent decapping in PBs (Kawa and Testerink, 2017), evidence from yeast suggests this link is not absolute for all mRNAs (Audebert et al., 2024). Similarly, although shorter poly(A) tails are generally correlated with reduced translation, a recent study in yeast proposed that deadenylation might primarily promote mRNA turnover rather than significantly affecting translation itself (Huch and Nissan, 2014). A further layer of translational control involves the miRNA pathway. In *A. thaliana*, Argonaute proteins (e.g., AGO1), loaded with miRNAs or small interfering RNAs, guide the silencing complex to target mRNAs (Baumberger and Baulcombe, 2005). There is strong evidence for AGO1 localizing within plant PBs (Pomeranz et al., 2010a). The accumulation of 3′-end fragments from miRNA-directed cleavage in *XRN4* mutants suggests that AGO1-mediated slicing often precedes 5′→3′ decay by XRN4, a process potentially facilitated by the co-localization of these factors in PBs (Rymarquis et al., 2011). The biogenesis of miRNAs itself involves other condensates, such as D-bodies, from which mature miRNAs are incorporated into AGO1 (Xie et al., 2021; Li et al., 2024). However, it remains an open question whether

schematic model; it does not imply that all decay occurs inside PBs. PB-localized and diffuse cytoplasmic decay likely coexist, and the relative contribution of each route is context dependent. NMD, nonsense-mediated decay; NGD, no-go decay; NSD, non-stop decay; DCP1, mRNA-decapping enzyme subunit 1; DCP2, mRNA-decapping enzyme subunit 2; DCP5, Protein decapping 5; EDC4/VCS, Enhancer of mRNA-decapping 4; ,LSM1–7, Sm-like proteins 1–7; XRN4, 5′→3′ exoribonuclease 4; PAT1, Protein associated with topoisomerase I; PARN, poly(A)-specific ribonuclease; Dhh1, DEAD-box helicases; CCR4-NOT, deadenylase complex.

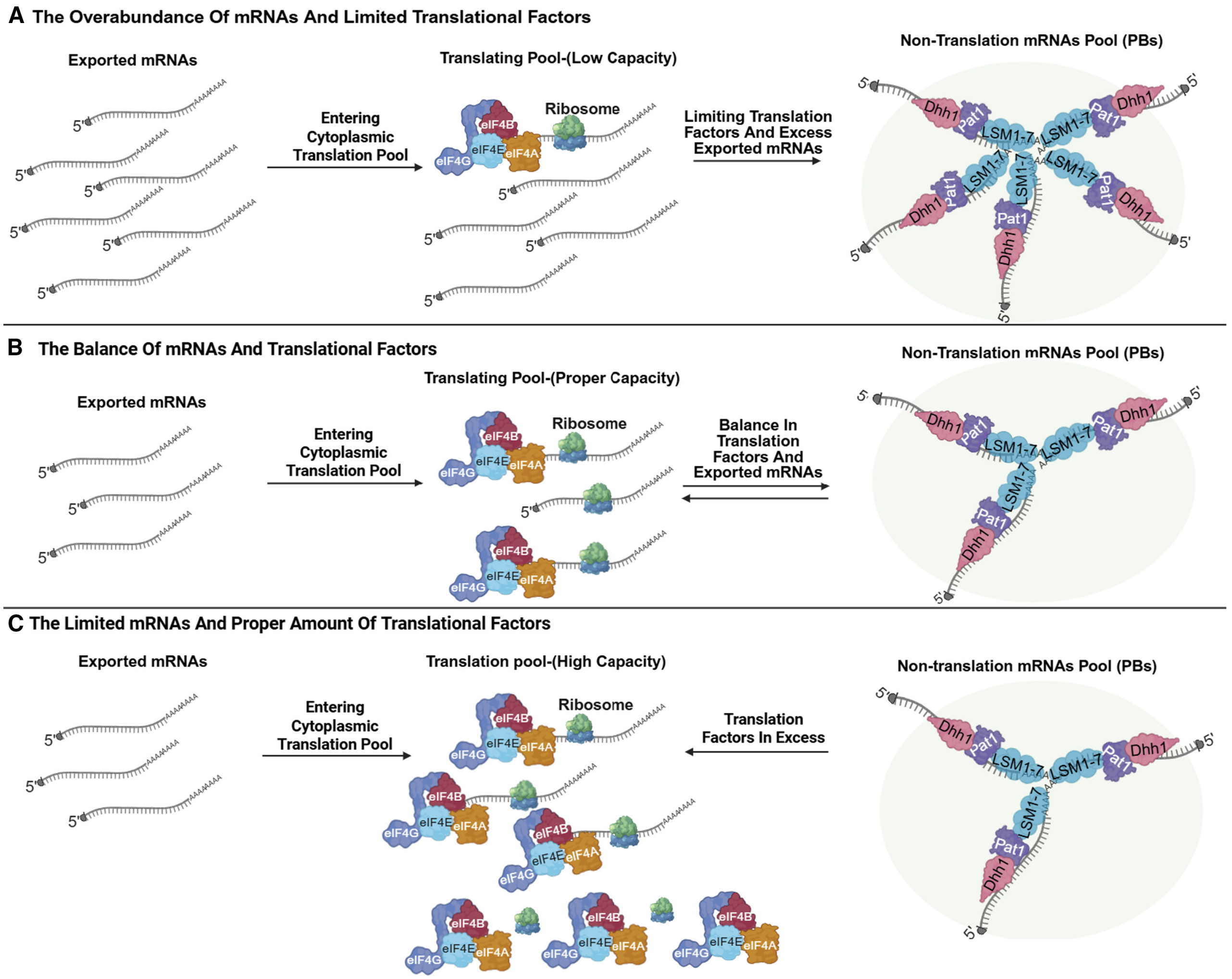

Figure 3. Conceptual model of PB buffering of cytoplasmic translation capacity and the pool of exported mRNAs.
(A) Limiting translation capacity with excess exported mRNAs. When exported mRNAs exceed available translation initiation factors and ribosomes, a larger fraction of transcripts transition into a non-translating state (often accompanied by deadenylation/oligoadenylation) and can associate with PB proteins (e.g., LSM1–7–PAT1 and Dhh1/DDX6), promoting PB assembly.
(B) Balanced supply. When translation capacity and exported mRNA supply are balanced, an equilibrium is maintained between translating mRNAs and a reversible pool of non-translating mRNPs transiently enriched in PBs.
(C) Translation factors in excess. When translation factors exceed available mRNAs, most transcripts remain in the translating pool, and PB-enriched transcripts may be released for re-entry into translation.
Note: this scheme summarizes an integrated working model based on published evidence; it does not imply that PBs are the exclusive site of translational repression or decay under all conditions. ,LSM1–7, Sm-like proteins 1–7; PAT1, Protein associated with topoisomerase I; Dhh1, DEAD-box helicases (e.g., RH6/RH8/RH12 in plants); eIF4A, Eukaryotic translation initiation factor 4A; eIF4B, Eukaryotic translation initiation factor 4B; eIF4G, Eukaryotic translation initiation factor 4G; eIF4E, Eukaryotic translation initiation factor 4E. See Coller and Parker (2004) and Maldonado-Bonilla (2014).

miRNA-targeted mRNAs merely pause within PBs or these structures are strictly required for efficient miRNA-mediated translational inhibition in plants (Xu and Chua, 2011) (Figure 2, black pathway; Table 1).

Role of plant PBs in stress responses

In contrast to mammals, where PB functions are often discussed in the context of neuronal plasticity and antiviral immunity, plant PBs act as central integrators of stress-response signaling, with prominent inputs from ABA and MAPK pathways during abiotic challenges such as drought and biotic stress, including pathogen attack (Yu et al., 2019; He et al., 2024). Owing to their sessile lifestyle, plants rely on sophisticated post-transcriptional mechanisms to cope with fluctuating environments. Under non-stress conditions, PBs help maintain RNA homeostasis during key developmental stages (e.g., seed germination and leaf growth). Upon stress, PBs typically increase in size and number, reflecting a global reorientation of cellular priorities toward survival (Huang et al., 2024). These dynamics are hormonally modulated, most notably via ABA, and their physiological relevance is underscored by severe phenotypes (including seedling lethality) in mutants of core PB components. Collectively, these features highlight the plant-specific tuning of PB function to physiological needs, distinguishing them from counterparts in other organisms (Maldonado-Bonilla, 2014).

Abiotic stress responses mediated by PBs

Abiotic factors such as drought, salinity, and heat disrupt cellular homeostasis, leading to the proliferation and functional reconfiguration of PBs. In *A. thaliana*, for example, heat stress (40°C) induces the clustering of DCP1 and DCP2 into visible PB-like granules, which readily disperse once the stress is removed (Motomura et al., 2015). PTMs of core PB components are a key mechanism for transducing stress signals. In *A. thaliana*, MAPK-dependent phosphorylation of DCP1 is a critical regulatory event during both abiotic and biotic stress. Dehydration, for instance, activates kinases such as MPK6 and SnRK2, which phosphorylate DCP1, modulating PB assembly and promoting selective mRNA decay to conserve cellular resources (Xu and Chua, 2012; Soma et al., 2017).

Furthermore, plant PBs serve as hubs for integrating hormonal signals, particularly ABA, to regulate mRNA fate. The LSM5/SAD1 protein, for example, enhances ABA sensitivity and promotes PB-mediated mRNA turnover during drought, as evidenced by developmental delays in *SAD1* mutants (Iwasaki et al., 2007; Maldonado-Bonilla, 2014). The functional integration of PB machinery with stress signaling is also exemplified by proteins such as TZF1; its overexpression enhances drought tolerance by modulating ABA-responsive transcripts, illustrating a unique plant adaptation (Maldonado-Bonilla, 2014).

Biotic stress responses and PB function

As mentioned earlier, the plant PBs frequently interact with SGs to coordinate the cellular response to stress. This interaction is particularly critical for adapting to abiotic stress (Chantarachot and Bailey-Serres, 2018). While similar crosstalk occurs in mammals, it is often associated with different contexts, such as immune responses or pathological states (Riggs et al., 2020). In mammalian cells, for instance, PB components such as LSM14A play a critical role in antiviral immune responses (Liu et al., 2016), establishing these condensates as hubs for pathogen defense.

The role of plant PBs during biotic stresses is an emerging field. While some viruses can manipulate PB components to favor their own replication cycle, as seen in other eukaryotes (Pérez-Vilaró et al., 2012; Lloyd, 2013; Reineke and Lloyd, 2013), PBs are also implicated in plant defense. Investigations into viral infections, such as those caused by tobacco mosaic virus (TMV), have shown elevated transcriptional levels of RNA decay factors, hinting at PB involvement in antiviral responses (Conti et al., 2017; Hoffmann et al., 2022; He et al., 2024). More direct evidence comes from studies on plant immunity. The protein TAF15b, for example, localizes to PBs during immune responses and is thought to regulate the stability of defense-related mRNAs (Dong et al., 2016). Similarly, the PB component VCS interacts with DCP5, and its overexpression enhances resistance to *Pseudomonas syringae*, boosting basal immunity (Panigrahi et al., 2024). On the other hand, recent evidence demonstrates that PBs can also be hijacked by pathogens or herbivores to suppress plant defense. For example, the aphid effector host-responsive cathepsin B (CathB6) associates with plant PBs and recruits key immune regulators, including enhanced disease susceptibility 1 (EDS1), phytoalexin deficient 4 (PAD4), and activated disease resistance 1 (ADR1), into these compartments, leading to repression of defense-related gene expression and attenuation of immune signaling. Thus, PBs function as regulatory hubs whose impact on plant immunity depends on whether they are engaged by endogenous regulatory pathways or exploited (Liu et al., 2025). These findings suggest that plant PBs, much like their mammalian counterparts, act as important arenas for host–pathogen interactions.

Although many studies have investigated PBs in mammals and yeast under stress or infection (Buchan et al., 2008; Reineke and Lloyd, 2013; van Leeuwen and Rabouille, 2019) and in human pathologies (Bhattacharyya et al., 2006; Riggs et al., 2020), their assembly under normal developmental conditions is less understood. For example, in plant seed development, mutants in PB components show significant developmental defects (Xu and Chua, 2011). Similar observations of PBs in non-stressed cells have been made in mouse fibroblasts, human HeLa cells, and yeast (Bashkirov et al., 1997; Ingelfinger et al., 2002; Borbolis et al., 2023), indicating that their formation is a constitutive aspect of cellular RNA metabolism. Overall, while PBs are not universally essential for mRNA decay, their dynamic assembly and context-dependent composition strongly suggest they function as critical regulatory hubs, fine-tuning post-transcriptional responses to both developmental and environmental signals.

Developmental roles of PBs across plant life stages

PBs are integral to post-transcriptional regulation throughout the plant life cycle. Crucially, genetic disruption of core PB components often leads to severe developmental phenotypes, particularly during key transitions such as seed germination, underscoring their essential role in clearing or storing specific mRNAs. For instance, in *A. thaliana*, PBs control the selective translation of almost 20% of mRNAs in dark-grown seedlings, a regulatory step crucial for optimizing the transition to photoautotrophic growth (Jang et al., 2019). Their functions are particularly prominent during seed germination and subsequent organ development.

Role in seed germination

During seed germination, PBs are crucial for managing mRNAs related to seed storage proteins (SSPs), such as oleosins and 12S/2S SSPs. Research indicates that, in wild-type *A. thaliana*, these mRNAs are translationally repressed and degraded to prevent unnecessary protein synthesis post germination. Evidence highlights that DCP5 is required for this repression. In the *DCP5-1* knockdown mutant, SSP mRNAs are translated, leading to significant accumulation of their products in 6-day-old germinated seedlings. This abnormal translation disrupts normal seedling development, underscoring the role of PB components in ensuring proper germination by clearing unnecessary mRNAs. Additionally, DCP5 and DCP1 accumulate during seed maturation, peaking in dry seeds, but decrease upon germination, while DCP2 is induced post germination, suggesting a dynamic assembly of PBs for mRNA storage and decapping during this transition (Xu and Chua, 2009). Consistent with this, mutations in the decapping complex proteins DCP1, DCP2, or VARICOSE in *A. thaliana* lead to severely retarded post-germination phenotypes (Kobayashi et al., 2007). Moreover, in light-grown *RH6*, *RH8*, and *RH12* mutants with reduced PBs, there is an increased level and translation of stress-related mRNAs under non-stress conditions, suggesting a role for PBs in repressing such transcripts during early growth (Chantarachot et al., 2020).

Role in leaf and root growth

PBs also influence postembryonic development, particularly in leaf and root growth, as mutants deficient in PB components exhibit severe developmental perturbations. For instance, *DCP5* and *VCS* mutants show abnormal leaves and vascular defects, indicating that PB-associated machinery regulates mRNAs critical for leaf morphology and vascular-system development (Maldonado-Bonilla, 2014). PBs also integrate hormonal signaling pathways into developmental control. In *A. thaliana*, XRN4 is allelic to Ethylene Insensitive 5 (EIN5). *XRN4* mutant seedlings exhibit ethylene insensitivity due to elevated levels of *EBF1* and *EBF2* mRNAs, which encode F-box proteins that target the key ethylene-responsive transcription factor EIN3 for degradation. Given that XRN4 localizes to PBs, these structures may serve as sites for modulating the ethylene signaling pathway, particularly during root development (Olmedo et al., 2006; Potuschak et al., 2006). Further support comes from *DCP5-1* mutants, which share developmental abnormalities with other decapping-deficient mutants, including pale and weak cotyledons (Xu and Chua, 2009). The *VCS* mutant also shows altered ABA responses, including reduced sensitivity to ABA-inhibited root growth, further linking PB function to hormonal control of development (Panigrahi et al., 2024).

Beyond organ growth, plant PBs also contribute to sharp cell-fate transitions. A recent study in *A. thaliana* showed that exit from meiosis requires PB-mediated translational repression of specific transcripts, and that disrupting core PB components delays or perturbs the meiotic-to-post-meiotic transition (Cairo et al., 2022). These data align with reports in animal systems where PB-like condensates modulate gene expression during differentiation and reprogramming, reinforcing the view that PBs act as regulatory hubs during developmental switches rather than only in steady-state growth.

AN INTERDISCIPLINARY FUTURE: INTEGRATING ADVANCED TOOLS TO DISSECT PLANT PB NETWORKS

Plant systems provide a unique opportunity to study PBs in a developmental and organismal context, enabling analysis of PB dynamics across cell-fate transitions and environmental responses (Field et al., 2023). Research on PBs has surged forward thanks to cutting-edge imaging technologies and high-throughput omics methods that collectively deepen our understanding of how these biomolecular condensates form, function, and adapt. Recent innovations have enabled precise visualization of PB formation, protein localization, and mRNA interactions in living cells (Xu and Chua, 2009; Steffens et al., 2014; Hoffmann et al., 2022). In parallel, proteomic and transcriptomic approaches have expanded our insight into the specific proteins and RNAs that reside in or transit through PBs, revealing key regulatory networks that underlie mRNA decay, translation repression, and stress adaptation (Kershaw et al., 2021).

Advanced imaging techniques for investigating PBs

Imaging breakthroughs have revolutionized PB research by enabling high-resolution, real-time visualization of these compartments in living cells. Approaches such as confocal and super-resolution microscopy offer detailed insights into this structure, assembly, and protein–mRNA localization (Hyjek-Składanowska et al., 2020). To date, confocal microscopy has been widely employed to visualize PBs in numerous studies (Goeres et al., 2007; Xu and Chua, 2009; Chantarachot et al., 2020; Liu et al., 2025). Techniques such as fluorescence recovery after photobleaching illustrate the mobility and turnover of PB constituents (Aizer et al., 2008). Additionally, fluorescence resonance energy transfer has proved invaluable for dissecting protein–protein and protein–RNA interactions that drive PB function (Andrei et al., 2005). Proximity ligation assay (PLA) is a method that detects close-proximity interactions between proteins, including those within PBs, with high sensitivity. PLA with accuracy below ∼40 nm has been particularly valuable for visualizing protein–protein interactions *in situ*, making it an ideal tool for studying PB assembly under varying conditions (Alam, 2018). A study demonstrated that plant PBs are not only cytoplasmic, membrane-less condensates involved in mRNA regulation but can also interface with the plasma membrane. Using proximity-biotinylation proteomics combined with *in situ* protein–protein interaction assays, including PLA and fluorescence resonance energy transfer, the authors show that the conserved PB component DCP1 localizes to specific plasma-membrane subdomains at cell edges and vertices. At these sites, DCP1 interacts with the suppressor of the cyclic AMP receptor (SCAR)–WASP family verprolin homologous (WAVE) complex independently of its decapping function, promoting localized actin nucleation and controlling cell shape and directional growth. Overall, this work revealed an unexpected, noncanonical role of PB components as spatial organizers of developmental processes (Liu et al., 2023). Cryoelectron microscopy offers three-dimensional imaging of PBs in near-native states, capturing their functional organization in response to environmental or metabolic signals (Danev et al., 2019). By combining these imaging methodologies, researchers can track PB formation, disassembly, and internal architecture with unprecedented clarity, laying the groundwork for understanding how PBs flexibly adapt to shifting demands on RNA metabolism.

Omics approaches to characterizing PB components

Alongside advanced microscopy, omics technologies have unlocked a more comprehensive perspective on PB composition and functions (Kershaw et al., 2021). In particular, proteomics employing high-throughput mass spectrometry enables the identification and quantification of PB-associated proteins, many of which participate in mRNA decapping, deadenylation, translation inhibition, or stress responses. These protein networks can then be mapped to reveal how post-transcriptional regulators converge within PBs to govern RNA stability and translation (Ayache et al., 2015; Hubstenberger et al., 2017). In parallel, transcriptomic analyses (e.g., RNA sequencing and single-cell RNA sequencing) provide insight into the RNA cargo captured or degraded in PBs under specific conditions. By profiling the mRNAs that accumulate, degrade, or shuttle to and from PBs, researchers can determine how PBs select certain transcripts for storage or decay (Matheny et al., 2019). For example, integrative multiomics analysis revealed that human PBs could repress the translation of the epithelial–mesenchymal transition (EMT) driver gene High Mobility Group AT-hook 2 (HMGA2), which contributed to

PB-mediated regulation of EMT (Fang et al., 2024). Beyond identifying the core PB proteome and transcriptome, recent studies underscore the significance of PTMs in modulating PB architecture and activity (Sternburg et al., 2022). Ongoing research aims to map the full repertoire of PTMs that trigger or restrain PB remodeling, thereby shedding light on new therapeutic strategies for diseases involving dysregulated RNA metabolism (Jeon et al., 2022).

Genetic dissection of PBs via CRISPR and phenotypic analysis

Understanding the PB composition and function requires precise manipulation of the genes encoding their constituent proteins, where CRISPR–Cas9 becomes invaluable. For instance, the combination of live-cell imaging with CRISPR-based genetics has been instrumental in dissecting protein functions *in vivo*. These methods, alongside live-cell single-molecule imaging for spatial decay kinetics, proximity labeling plus quantitative proteomics for context-specific composition, CRISPR allelic series for causality, and computational integration (from motif discovery to machine learning based condensate-propensity predictors) (Dhamodharan et al., 2022; Brothers et al., 2023). For example, knockdown of DCP5 in *A. thaliana*, combined with live-cell imaging, revealed its dual role in PB assembly and nuclear transcriptional control at FLOWERING LOCUS C (FLC) (Wang et al., 2023). In addition, in a study on *Candida albicans*, CRISPR–Cas9 was used to delete genes DHH1 and EDC3, which are linked to PBs. The results showed that PBs can still form during heat shock even without DHH1 or EDC3, suggesting these proteins are not always needed. The phenotypic analysis revealed that only completely removing DHH1 (not just reducing it) affects how the fungus grows in a thread-like way, fixing past misunderstandings. Additionally, removing DHH1 altered gene expression related to growth and stress, highlighting its role in PBs for controlling genes. This shows CRISPR's precision was key in getting clear, reliable insights into PB functions (Tosiano et al., 2025). The study on humans demonstrates that 5-diphosphoinositol pentakisphosphate (5-InsP7) directly inhibits the decapping activity of NUDT3 *in vitro*, using recombinant NUDT3 to show this effect in a controlled setting. This inhibition was further confirmed in intact cells using genetic manipulation and pharmacological approaches, specifically in HEK293 and HCT116 cell lines. Diphosphoinositol pentakisphosphate 5-kinases type 1 and 2 (PPIP5Ks) are enzymes that convert 5-InsP7 to InsP8, a higher-order inositol pyrophosphate. Knocking out PPIP5Ks using CRISPR–Cas9 increases cellular 5-InsP7 levels by two- to three-fold, within the physiological rheostatic range. This elevation leads to an increased abundance of PBs, paralleling the stabilization of mRNAs (Sahu et al., 2020). As another example, a pooled CRISPR screen targeting RBPs identified core PB components, including DDX6 and EDC4, as negative regulators of cell migration. Loss of these PB components promoted EMT through translational repression of specific mRNAs, revealing a direct role for PBs in post-transcriptional gene regulation (Fang et al., 2024). A recent study used genome-wide CRISPR screens to identify PB regulators as critical vulnerabilities in acute myeloid leukemia. The study showed that leukemia cells have abnormally elevated numbers of PBs, which are essential for acute myeloid leukemia initiation and maintenance, while their loss had minimal impact on normal hematopoiesis but disrupted regenerative hematopoiesis (Kodali et al., 2024). While the use of CRISPR in plant PB investigation remains in its early stages, the technique's precision and versatility offer exciting potential. CRISPR could significantly advance our understanding of plant PBs and unlock new opportunities for agricultural innovation, cell homeostasis, and stress-resilience research. There are several ways in which CRISPR could provide valuable insights into plant PB biology''

- Avoiding off-target effects: traditional methods, such as random mutagenesis or less specific deletion techniques, often lead to unintended genetic changes, complicating the interpretation of phenotypic outcomes. CRISPR–Cas9 minimizes these issues, ensuring that observed effects are due to the targeted gene modification.
- Studying individual components: PBs involve numerous proteins, and their interactions are complex. CRISPR allows researchers to knock out or modify one gene at a time, isolating its role in PB assembly, mRNA decay, or other functions. For instance, it can help determine whether a protein is essential for PB condensation or has additional cytoplasmic roles.
- Facilitating functional genomics: by generating precise mutants, CRISPR enables functional genomic studies, such as transcriptome analysis, to understand how PB-related genes influence global gene expression. This is particularly relevant for understanding how PBs regulate cellular processes such as stress response and morphogenesis.
- Endogenous protein tagging: CRISPR could be used to insert fluorescent tags into genes encoding PB proteins. Tagging endogenous proteins avoids the overexpression artifacts that can occur with traditional fluorescent protein tagging methods, leading to more accurate observations. This approach would enable real-time imaging of PB dynamics in living plant cells, providing insights into how these structures assemble, disassemble, and respond to environmental stimuli or developmental conditions.

Applying CRISPR in plant PB research not only promises to clarify the role of these structures in mRNA metabolism but may also reveal plant-specific mechanisms that differ from those in animal cells. This could broaden our overall understanding of cellular regulation and RNA dynamics.

CONCLUSIONS AND PERSPECTIVES

Over the past two decades, research in plant models such as *A. thaliana*, *Nicotiana tabacum*, *Nicotiana benthamiana*, *Brassica napus*, *Hylocereus polyrhizus*, wheat, and rice has delivered remarkable insights into the structural and functional dynamics of PBs (Table 2). While sharing a conserved core with their yeast and mammalian counterparts, plant PBs have evolved unique components, such as DCP5, and are subject to regulatory inputs from hormonal and environmental signaling pathways. This review synthesizes the current, plant-focused understanding of their composition; LLPS-driven assembly; and multifaceted roles in mRNA decay, storage, and stress responses.

However, significant questions remain. The future of plant PB research is inherently interdisciplinary, perfectly aligning with the modern direction of plant science that integrates genomics,

quantitative proteomics, advanced imaging, and bioinformatics. Addressing these questions will be essential, and three key areas demand further investigation:

1. What are the kinetics of mRNA decay within PBs versus the cytoplasm in plant cells?
While PBs concentrate decapping and decay machinery, the spatial and temporal dynamics of mRNA degradation within these condensates remain poorly resolved in plants. Do PBs function primarily as sites of active decay, or do they serve as temporary storage depots where mRNAs are sequestered for later degradation or re-translation? Advanced imaging techniques, such as single-molecule fluorescence *in situ* hybridization combined with live-cell microscopy, could provide real-time insights into the compartmentalization of decay processes.

2. How do PTMs regulate PB dynamics under stress?
PTMs are emerging as pivotal regulators of PB assembly and function. In plants, MAPK-dependent phosphorylation of DCP1 during abiotic and biotic stress modulates PB formation, yet the downstream effects on mRNA targeting and decay remain unclear. For instance, how does phosphorylation of core components alter the recruitment of specific mRNAs or the interaction with SGs? Systematic proteomic analyses of PTMs under varying stress conditions, combined with CRISPR-based mutagenesis of phosphorylation sites, could elucidate these mechanisms and reveal whether other modifications, such as ubiquitination, play a similar role, as observed in mammalian systems.

3. What is the molecular basis for selective mRNA targeting to PBs in plants?
The mechanisms governing the selective recruitment of mRNAs to PBs are still largely unknown. Recent evidence suggests that RNA modifications, such as m^6A, and specific RBPs (e.g., ECT8, TZF family proteins) contribute to this process. For example, the m^6A reader ECT8 interacts with DCP5 to promote the decay of stress-responsive transcripts during salt stress. High-throughput approaches, such as RNA sequencing of PB-associated transcripts, are needed to identify the full range of *cis*- and *trans*-acting factors that dictate mRNA sorting.

Answering these fundamental questions not only deepens our understanding of gene regulation but also holds significant translational potential. Together with recent work on meiotic exit, these findings highlight PBs as key regulators of cell-fate transitions in plants and not just as housekeeping hubs for bulk mRNA turnover. A clearer grasp of how PBs modulate plant development and stress responses could pave the way for engineering stress-resilient crops by enabling the targeted manipulation of PB components to fine-tune the stability of mRNAs critical for growth and stress adaptation, thereby bolstering agricultural productivity and sustainability in the face of climate change.

FUNDING
This work was supported by the Polish National Science Centre (UMO-2022/45/N/NZ3/02015, UMO-2023/49/B/NZ3/03815, and UMO-2025/57/B/NZ3/04848) (K.M. and D.J.S.) and by "The Excellence Initiative – Research University" programme – Nicolaus Copernicus University (A.M., Z.Z., K.M., and D.J.S.).

ACKNOWLEDGMENTS
No conflict of interest is declared.

AUTHOR CONTRIBUTIONS
A.M. and D.J.S. conceptualized the review. A.M., Z.Z., K.M., and D.J.S. drafted the manuscript. A.M., Z.Z., K.M., and D.J.S. revised the manuscript. All authors discussed the content and approved the final version.

SUPPLEMENTAL INFORMATION
Supplemental information is available at *Plant Communications Online*.

Received: September 23, 2025
Revised: November 1, 2025
Accepted: February 26, 2026
Published: March 3, 2026

REFERENCES
Aizer, A., and Shav-Tal, Y. (2008). Intracellular trafficking and dynamics of P bodies. Prion **2**:131–134.

Aizer, A., Brody, Y., Ler, L.W., Sonenberg, N., Singer, R.H., and Shav-Tal, Y. (2008). The dynamics of mammalian P body transport, assembly, and disassembly in vivo. Mol. Biol. Cell **19**:4154–4166.

Aizer, A., Kafri, P., Kalo, A., and Shav-Tal, Y. (2013). The P body protein Dcp1a is hyper-phosphorylated during mitosis. PLoS One **8**:e49783.

Aizer, A., Kalo, A., Kafri, P., Shraga, A., Ben-Yishay, R., Jacob, A., Kinor, N., and Shav-Tal, Y. (2014). Quantifying mRNA targeting to P-bodies in living human cells reveals their dual role in mRNA decay and storage. J. Cell Sci. **127**:4443–4456.

Alam, M.S. (2018). Proximity ligation assay (PLA). Curr. Protoc. Immunol. **123**:e58.

Alberti, S., Gladfelter, A., and Mittag, T. (2019). Considerations and challenges in studying liquid-liquid phase separation and biomolecular condensates. Cell **176**:419–434.

Andrei, M.A., Ingelfinger, D., Heintzmann, R., Achsel, T., Rivera-Pomar, R., and Lührmann, R. (2005). A role for eIF4E and eIF4E-transporter in targeting mRNPs to mammalian processing bodies. Rna **11**:717–727.

Audebert, L., Feuerbach, F., Zedan, M., Schürch, A.P., Decourty, L., Namane, A., Permal, E., Weis, K., Badis, G., and Saveanu, C. (2024). RNA degradation triggered by decapping is largely independent of initial deadenylation. EMBO J. **43**:6496–6524.

Ayache, J., Bénard, M., Ernoult-Lange, M., Minshall, N., Standart, N., Kress, M., and Weil, D. (2015). P-body assembly requires DDX6 repression complexes rather than decay or Ataxin2/2L complexes. Mol. Biol. Cell **26**:2579–2595.

Bah, A., and Forman-Kay, J.D. (2016). Modulation of intrinsically disordered protein function by post-translational modifications. J. Biol. Chem. **291**:6696–6705.

Balagopal, V., and Parker, R. (2009). Polysomes, P bodies and stress granules: states and fates of eukaryotic mRNAs. Curr. Opin. Cell Biol. **21**:403–408.

Bashkirov, V.I., Scherthan, H., Solinger, J.A., Buerstedde, J.M., and Heyer, W.D. (1997). A mouse cytoplasmic exoribonuclease (mXRN1p) with preference for G4 tetraplex substrates. J. Cell Biol. **136**:761–773.

Baumberger, N., and Baulcombe, D.C. (2005). Arabidopsis ARGONAUTE1 is an RNA Slicer that selectively recruits microRNAs

and short interfering RNAs. Proc. Natl. Acad. Sci. USA **102**:11928–11933.

Bhattacharyya, S.N., Habermacher, R., Martine, U., Closs, E.I., and Filipowicz, W. (2006). Stress-induced reversal of microRNA repression and mRNA P-body localization in human cells. Cold Spring Harb. Symp. Quant. Biol. **71**:513–521.

Bhullar, D.S., Sheahan, M.B., and Rose, R.J. (2017). RNA processing body (P-body) dynamics in mesophyll protoplasts re-initiating cell division. Protoplasma **254**:1627–1637.

Blake, L.A., Watkins, L., Liu, Y., Inoue, T., and Wu, B. (2024). A rapid inducible RNA decay system reveals fast mRNA decay in P-bodies. Nat. Commun. **15**:2720.

Bogamuwa, S., and Jang, J.C. (2016). Plant tandem CCCH zinc finger proteins interact with ABA, drought, and stress response regulators in processing-bodies and stress granules. PLoS One **11**:e0151574.

Borbolis, F., Ranti, D., Papadopoulou, M.D., Dimopoulou, S., Malatras, A., Michalopoulos, I., and Syntichaki, P. (2023). Selective destabilization of transcripts by mRNA decapping regulates oocyte maturation and innate immunity gene expression during ageing in C. elegans. Biology **12**:171.

Brengues, M., and Parker, R. (2007). Accumulation of polyadenylated mRNA, Pab1p, eIF4E, and eIF4G with P-bodies in Saccharomyces cerevisiae. Mol. Biol. Cell **18**:2592–2602.

Brengues, M., Teixeira, D., and Parker, R. (2005). Movement of eukaryotic mRNAs between polysomes and cytoplasmic processing bodies. Science **310**:486–489.

Brogna, S., and Wen, J. (2009). Nonsense-mediated mRNA decay (NMD) mechanisms. Nat. Struct. Mol. Biol. **16**:107–113.

Brothers, W.R., Ali, F., Kajjo, S., and Fabian, M.R. (2023). The EDC4-XRN1 interaction controls P-body dynamics to link mRNA decapping with decay. EMBO J. **42**:EMBJ2023113933.

Buchan, J.R. (2024). Stress granule and P-body clearance: Seeking coherence in acts of disappearance. Semin. Cell Dev. Biol. **159–160**:10–26.

Buchan, J.R., Muhlrad, D., and Parker, R. (2008). P bodies promote stress granule assembly in Saccharomyces cerevisiae. J. Cell Biol. **183**:441–455.

Burke, K.A., Janke, A.M., Rhine, C.L., and Fawzi, N.L. (2015). Residue-by-residue view of in vitro FUS granules that bind the C-terminal domain of RNA polymerase II. Mol. Cell **60**:231–241.

Cai, Z., Tang, Q., Song, P., Tian, E., Yang, J., and Jia, G. (2024). The m6A reader ECT8 is an abiotic stress sensor that accelerates mRNA decay in Arabidopsis. Plant Cell **36**:2908–2926.

Cairo, A., Vargova, A., Shukla, N., Capitao, C., Mikulkova, P., Valuchova, S., Pecinkova, J., Bulankova, P., and Riha, K. (2022). Meiotic exit in Arabidopsis is driven by P-body–mediated inhibition of translation. Science **377**:629–634.

Carpentier, M.C., Receveur, A.E., Boubegtitene, A., Cadoudal, A., Bousquet-Antonelli, C., and Merret, R. (2024). Genome-wide analysis of mRNA decay in Arabidopsis shoot and root reveals the importance of co-translational mRNA decay in the general mRNA turnover. Nucleic Acids Res. **52**:7910–7924.

Chan, S.P., and Slack, F.J. (2006). MicroRNA-mediated silencing inside P bodies. RNA Biol. **3**:97–100.

Chantarachot, T., and Bailey-Serres, J. (2018). Polysomes, stress granules, and processing bodies: a dynamic triumvirate controlling cytoplasmic mRNA fate and function. Plant Physiol. **176**:254–269.

Chantarachot, T., Sorenson, R.S., Hummel, M., Ke, H., Kettenburg, A.T., Chen, D., Aiyetiwa, K., Dehesh, K., Eulgem, T., Sieburth, L.E., et al. (2020). DHH1/DDX6-like RNA helicases maintain ephemeral half-lives of stress-response mRNAs. Nat. Plants **6**:675–685.

Chekanova, J.A., Dutko, J.A., Mian, I.S., and Belostotsky, D.A. (2002). Arabidopsis thaliana exosome subunit AtRrp4p is a hydrolytic 3′→ 5′ exonuclease containing S1 and KH RNA-binding domains. Nucleic Acids Res. **30**:695–700.

Chekanova, J.A., Gregory, B.D., Reverdatto, S.V., Chen, H., Kumar, R., Hooker, T., Yazaki, J., Li, P., Skiba, N., Peng, Q., et al. (2007). Genome-wide high-resolution mapping of exosome substrates reveals hidden features in the Arabidopsis transcriptome. Cell **131**:1340–1353.

Chen, C.Y.A., and Shyu, A.B. (2011). Mechanisms of deadenylation-dependent decay. Wiley Interdiscip. Rev. RNA **2**:167–183.

Chodasiewicz, M., Sokolowska, E.M., Nelson-Dittrich, A.C., Masiuk, A., Beltran, J.C.M., Nelson, A.D.L., and Skirycz, A. (2020). Identification and characterization of the heat-induced plastidial stress granules reveal new insight into Arabidopsis stress response. Front. Plant Sci. **11**:595792.

Chou, W.L., Huang, L.F., Fang, J.C., Yeh, C.H., Hong, C.Y., Wu, S.J., and Lu, C.A. (2014). Divergence of the expression and subcellular localization of CCR4-associated factor 1 (CAF1) deadenylase proteins in Oryza sativa. Plant Mol. Biol. **85**:443–458.

Coller, J., and Parker, R. (2004). Eukaryotic mRNA decapping. Annu. Rev. Biochem. **73**:861–890.

Conti, G., Zavallo, D., Venturuzzi, A.L., Rodriguez, M.C., Crespi, M., and Asurmendi, S. (2017). TMV induces RNA decay pathways to modulate gene silencing and disease symptoms. Plant J. **89**:73–84.

Covarrubias, A.A., and Reyes, J.L. (2010). Post-transcriptional gene regulation of salinity and drought responses by plant microRNAs. Plant Cell Environ. **33**:481–489.

Crisp, P.A., Ganguly, D.R., Smith, A.B., Murray, K.D., Estavillo, G.M., Searle, I., Ford, E., Bogdanović, O., Lister, R., Borevitz, J.O., et al. (2017). Rapid recovery gene downregulation during excess-light stress and recovery in Arabidopsis. Plant Cell **29**:1836–1863.

Dai, Y., Li, W., and An, L. (2016). NMD mechanism and the functions of Upf proteins in plant. Plant Cell Rep. **35**:5–15.

Danev, R., Yanagisawa, H., and Kikkawa, M. (2019). Cryo-electron microscopy methodology: current aspects and future directions. Trends Biochem. Sci. **44**:837–848.

Decker, C.J., and Parker, R. (2012). P-bodies and stress granules: possible roles in the control of translation and mRNA degradation. Cold Spring Harb. Perspect. Biol. **4**:a012286.

Dhamodharan, A., Okada, H., Muraoka, M., Wright, D., Okubo, A., and Saga, Y. (2022). P-body dynamics revealed by DDX6 protein knockdown via the auxin-inducible degron system. Dev. Growth Differ. **64**:537–547.

Dong, O.X., Meteignier, L.V., Plourde, M.B., Ahmed, B., Wang, M., Jensen, C., Jin, H., Moffett, P., Li, X., and Germain, H. (2016). Arabidopsis TAF15b localizes to RNA processing bodies and contributes to snc1-mediated autoimmunity. Mol. Plant Microbe Interact. **29**:247–257.

Elbaum-Garfinkle, S., Kim, Y., Szczepaniak, K., Chen, C.C.H., Eckmann, C.R., Myong, S., and Brangwynne, C.P. (2015). The disordered P granule protein LAF-1 drives phase separation into droplets with tunable viscosity and dynamics. Proc. Natl. Acad. Sci. USA **112**:7189–7194.

Eulalio, A., Behm-Ansmant, I., and Izaurralde, E. (2007a). P bodies: at the crossroads of post-transcriptional pathways. Nat. Rev. Mol. Cell Biol. **8**:9–22.

Eulalio, A., Behm-Ansmant, I., Schweizer, D., and Izaurralde, E. (2007b). P-body formation is a consequence, not the cause, of RNA-mediated gene silencing. Mol. Cell Biol. **27**:3970–3981.

Fang, L., Zhang, L., Wang, M., He, Y., Yang, J., Huang, Z., Tan, Y., Fang, K., Li, J., Sun, Z., et al. (2024). Pooled CRISPR Screening Identifies P-Bodies as Repressors of Cancer Epithelial–Mesenchymal Transition. Cancer Res. **84**:659–674.

Feric, M., Vaidya, N., Harmon, T.S., Mitrea, D.M., Zhu, L., Richardson, T.M., Kriwacki, R.W., Pappu, R.V., and Brangwynne, C.P. (2016). Coexisting liquid phases underlie nucleolar subcompartments. Cell **165**:1686–1697.

Field, S., Jang, G.J., Dean, C., Strader, L.C., and Rhee, S.Y. (2023). Plants use molecular mechanisms mediated by biomolecular condensates to integrate environmental cues with development. Plant Cell **35**:3173–3186.

Flemr, M., Ma, J., Schultz, R.M., and Svoboda, P. (2010). P-body loss is concomitant with formation of a messenger RNA storage domain in mouse oocytes. Biol. Reprod. **82**:1008–1017.

Forbes Beadle, L., Love, J.C., Shapovalova, Y., Artemev, A., Rattray, M., and Ashe, H.L. (2023). Combined modelling of mRNA decay dynamics and single-molecule imaging in the Drosophila embryo uncovers a role for P-bodies in 5′ to 3′ degradation. PLoS Biol. **21**:e3001956.

Foressi, N.N., Cruz Rodríguez, L., Wilke, N., and Celej, M.S. (2025). Cation-driven modulation of tau condensates: insights into liquid–liquid phase separation and rheological properties. Biomacromolecules **26**:3605–3616.

Franks, T.M., and Lykke-Andersen, J. (2008). The control of mRNA decapping and P-body formation. Mol. Cell **32**:605–615.

Fu, Y., Dominissini, D., Rechavi, G., and He, C. (2014). Gene expression regulation mediated through reversible m6A RNA methylation. Nat. Rev. Genet. **15**:293–306.

García, R., Pulido, V., Orellana-Muñoz, S., Nombela, C., Vázquez de Aldana, C.R., Rodríguez-Peña, J.M., and Arroyo, J. (2019). Signalling through the yeast MAPK Cell Wall Integrity pathway controls P-body assembly upon cell wall stress. Sci. Rep. **9**:3186.

Garneau, N.L., Wilusz, J., and Wilusz, C.J. (2007). The highways and byways of mRNA decay. Nat. Rev. Mol. Cell Biol. **8**:113–126.

Goeres, D.C., Van Norman, J.M., Zhang, W., Fauver, N.A., Spencer, M.L., and Sieburth, L.E. (2007). Components of the Arabidopsis mRNA decapping complex are required for early seedling development. Plant Cell **19**:1549–1564.

Gudkova, D.O., Panasyuk, G.G., Nemazanyy, I.O., and Filonenko, V.V. (2011). PI3K/mTOR-dependent signaling pathway as a possible regulator of processing body assembly. Biopolym. Cell **27**:369–372.

Gutierrez-Beltran, E., Moschou, P.N., Smertenko, A.P., and Bozhkov, P.V. (2015). Tudor staphylococcal nuclease links formation of stress granules and processing bodies with mRNA catabolism in Arabidopsis. Plant Cell **27**:926–943.

Guzikowski, A.R., Chen, Y.S., and Zid, B.M. (2019). Stress-induced mRNP granules: Form and function of processing bodies and stress granules. Wiley Interdiscip. Rev. RNA **10**:e1524.

Hamada, T., Yako, M., Minegishi, M., Sato, M., Kamei, Y., Yanagawa, Y., Toyooka, K., Watanabe, Y., and Hara-Nishimura, I. (2018). Stress granule formation is induced by a threshold temperature rather than a temperature difference in Arabidopsis. J. Cell Sci. **131**:jcs216051.

Han, W.Y., Hou, B.H., Lee, W.C., Chan, T.C., Lin, T.H., and Chen, H.M. (2023). Arabidopsis mRNA decay landscape shaped by XRN 5′-3′ exoribonucleases. Plant J. **114**:895–913.

He, H., Ge, L., Chen, Y., Zhao, S., Li, Z., Zhou, X., and Li, F. (2024). m6A modification of plant virus enables host recognition by NMD factors in plants. Sci. China Life Sci. **67**:161–174.

He, S.L., Wang, X., Kim, S.I., Kong, L., Liu, A., Wang, L., Wang, Y., Shan, L., He, P., and Jang, J.C. (2024). Modulation of stress granule dynamics by phosphorylation and ubiquitination in plants. iScience **27**:111162.

Hilliker, A. (2012). Analysis of RNA helicases in P-bodies and stress granules. Methods Enzymol. **511**:323–346.

Hoffmann, G., Mahboubi, A., Bente, H., Garcia, D., Hanson, J., and Hafrén, A. (2022). Arabidopsis RNA processing body components LSM1 and DCP5 aid in the evasion of translational repression during Cauliflower mosaic virus infection. Plant Cell **34**:3128–3147.

Hofweber, M., and Dormann, D. (2019). Friend or foe—Post-translational modifications as regulators of phase separation and RNP granule dynamics. J. Biol. Chem. **294**:7137–7150.

Holehouse, A.S., and Pappu, R.V. (2018). Functional implications of intracellular phase transitions. Biochemistry **57**:2415–2423.

Horvathova, I., Voigt, F., Kotrys, A.V., Zhan, Y., Artus-Revel, C.G., Eglinger, J., Stadler, M.B., Giorgetti, L., and Chao, J.A. (2017). The dynamics of mRNA turnover revealed by single-molecule imaging in single cells. Mol. Cell **68**:615–625.e9.

Hou, C.Y., Lee, W.C., Chou, H.C., Chen, A.P., Chou, S.J., and Chen, H.M. (2016). Global analysis of truncated RNA ends reveals new insights into ribosome stalling in plants. Plant Cell **28**:2398–2416.

Hoyle, N.P., Castelli, L.M., Campbell, S.G., Holmes, L.E.A., and Ashe, M.P. (2007). Stress-dependent relocalization of translationally primed mRNPs to cytoplasmic granules that are kinetically and spatially distinct from P-bodies. J. Cell Biol. **179**:65–74.

Huang, Z., Xu, Z., Liu, X., Chen, G., Hu, C., Chen, M., and Liu, Y. (2024). Exploring the role of the processing body in plant abiotic stress response. Curr. Issues Mol. Biol. **46**:9844–9855.

Hubstenberger, A., Courel, M., Bénard, M., Souquere, S., Ernoult-Lange, M., Chouaib, R., Yi, Z., Morlot, J.B., Munier, A., Fradet, M., et al. (2017). P-body purification reveals the condensation of repressed mRNA regulons. Mol. Cell **68**:144–157.e5.

Huch, S., and Nissan, T. (2014). Interrelations between translation and general mRNA degradation in yeast. Wiley Interdiscip. Rev. RNA **5**:747–763.

Hyjek-Składanowska, M., Bajczyk, M., Gołębiewski, M., Nuc, P., Kołowerzo-Lubnau, A., Jarmołowski, A., and Smoliński, D.J. (2020). Core spliceosomal Sm proteins as constituents of cytoplasmic mRNPs in plants. Plant J. **103**:1155–1173.

Ingelfinger, D., Arndt-Jovin, D.J., Lührmann, R., and Achsel, T. (2002). The human LSm1-7 proteins colocalize with the mRNA-degrading enzymes Dcp1/2 and Xrn1 in distinct cytoplasmic foci. Rna **8**:1489–1501.

Ismail, H., Liu, X., Yang, F., Li, J., Zahid, A., Dou, Z., Liu, X., and Yao, X. (2021). Mechanisms and regulation underlying membraneless organelle plasticity control. J. Mol. Cell Biol. **13**:239–258.

Iwasaki, S., Takeda, A., Motose, H., and Watanabe, Y. (2007). Characterization of Arabidopsis decapping proteins AtDCP1 and AtDCP2, which are essential for post-embryonic development. FEBS Lett. **581**:2455–2459.

Jackson, R.J., Hellen, C.U.T., and Pestova, T.V. (2012). Termination and post-termination events in eukaryotic translation. Adv. Protein Chem. Struct. Biol. **86**:45–93.

Jan, A., Maruyama, K., Todaka, D., Kidokoro, S., Abo, M., Yoshimura, E., Shinozaki, K., Nakashima, K., and Yamaguchi-Shinozaki, K. (2013). OsTZF1, a CCCH-tandem zinc finger protein, confers delayed senescence and stress tolerance in rice by regulating stress-related genes. Plant Physiol. **161**:1202–1216.

Jang, G.J., Yang, J.Y., Hsieh, H.L., and Wu, S.H. (2019). Processing bodies control the selective translation for optimal development of Arabidopsis young seedlings. Proc. Natl. Acad. Sci. USA **116**:6451–6456.

Jeon, P., Ham, H.J., Park, S., and Lee, J.A. (2022). Regulation of cellular ribonucleoprotein granules: from assembly to degradation via post-translational modification. Cells **11**:2063.

Jeong, H.J., Kim, Y.J., Kim, S.H., Kim, Y.H., Lee, I.J., Kim, Y.K., and Shin, J.S. (2011). Nonsense-mediated mRNA decay factors, UPF1 and UPF3, contribute to plant defense. Plant Cell Physiol. **52**:2147–2156.

Jonas, S., and Izaurralde, E. (2013). The role of disordered protein regions in the assembly of decapping complexes and RNP granules. Genes Dev. **27**:2628–2641.

Kawa, D., and Testerink, C. (2017). Regulation of mRNA decay in plant responses to salt and osmotic stress. Cell. Mol. Life Sci. **74**:1165–1176.

Kearly, A., Nelson, A.D.L., Skirycz, A., and Chodasiewicz, M. (2024). Composition and function of stress granules and P-bodies in plants. Semin. Cell Dev. Biol. **156**:167–175.

Kedersha, N., and Anderson, P. (2009). Regulation of translation by stress granules and processing bodies. Prog. Mol. Biol. Transl. Sci. **90**:155–185.

Kedersha, N., Stoecklin, G., Ayodele, M., Yacono, P., Lykke-Andersen, J., Fritzler, M.J., Scheuner, D., Kaufman, R.J., Golan, D.E., and Anderson, P. (2005). Stress granules and processing bodies are dynamically linked sites of mRNP remodeling. J. Cell Biol. **169**:871–884.

Kedia, S., Aghanoori, M.R., Burns, K.M.L., Subha, M., Williams, L., Wen, P., Kopp, D., Erickson, S.L., Harvey, E.M., Chen, X., et al. (2022). Ubiquitination and deubiquitination of 4E-T regulate neural progenitor cell maintenance and neurogenesis by controlling P-body formation. Cell Rep. **40**:111070.

Kershaw, C.J., Nelson, M.G., Lui, J., Bates, C.P., Jennings, M.D., Hubbard, S.J., Ashe, M.P., and Grant, C.M. (2021). Integrated multi-omics reveals common properties underlying stress granule and P-body formation. RNA Biol. **18**:655–673.

Kim, J., Lee, H., Lee, H.G., and Seo, P.J. (2021). Get closer and make hotspots: liquid–liquid phase separation in plants. EMBO Rep. **22**:EMBR202051656.

Kobayashi, K., Otegui, M.S., Krishnakumar, S., Mindrinos, M., and Zambryski, P. (2007). INCREASED SIZE EXCLUSION LIMIT2 encodes a putative DEVH box RNA helicase involved in plasmodesmata function during Arabidopsis embryogenesis. Plant Cell **19**:1885–1897.

Kodali, S., Proietti, L., Valcarcel, G., López-Rubio, A.V., Pessina, P., Eder, T., Shi, J., Jen, A., Lupión-Garcia, N., Starner, A.C., et al. (2024). RNA sequestration in P-bodies sustains myeloid leukaemia. Nat. Cell Biol. **26**:1745–1758.

Kong, W., Tan, S., Zhao, Q., Lin, D.L., Xu, Z.H., Friml, J., and Xue, H.W. (2021). mRNA surveillance complex PELOTA–HBS1 regulates phosphoinositide-dependent protein kinase1 and plant growth. Plant Physiol. **186**:2003–2020.

Lee, Y., Choe, J., Park, O.H., and Kim, Y.K. (2020). Molecular mechanisms driving mRNA degradation by m6A modification. Trends Genet. **36**:177–188.

Li, Q., Liu, Y., and Zhang, X. (2024). Biomolecular condensates in plant RNA silencing: insights into formation, function, and stress responses. Plant Cell **36**:227–245.

Li, Q., Shortreed, M.R., Wenger, C.D., Frey, B.L., Schaffer, L.V., Scalf, M., and Smith, L.M. (2017). Global post-translational modification discovery. J. Proteome Res. **16**:1383–1390.

Li, W., Ma, M., Feng, Y., Li, H., Wang, Y., Ma, Y., Li, M., An, F., and Guo, H. (2015). EIN2-directed translational regulation of ethylene signaling in Arabidopsis. Cell **163**:670–683.

Li, X., Li, C., Zhu, J., Zhong, S., Zhu, H., and Zhang, X. (2023). Functions and mechanisms of RNA helicases in plants. J. Exp. Bot. **74**:2295–2310.

Lin, Y., Protter, D.S.W., Rosen, M.K., and Parker, R. (2015). Formation and maturation of phase-separated liquid droplets by RNA-binding proteins. Mol. Cell **60**:208–219.

Ling, S.H.M., Qamra, R., and Song, H. (2011). Structural and functional insights into eukaryotic mRNA decapping. Wiley Interdiscip. Rev. RNA **2**:193–208.

Liu, C., Mentzelopoulou, A., Muhammad, A., Volkov, A., Weijers, D., Gutierrez-Beltran, E., and Moschou, P.N. (2023). An actin remodeling role for Arabidopsis processing bodies revealed by their proximity interactome. EMBO J. **42**:EMBJ2022111885.

Liu, J., Valencia-Sanchez, M.A., Hannon, G.J., and Parker, R. (2005). MicroRNA-dependent localization of targeted mRNAs to mammalian P-bodies. Nat. Cell Biol. **7**:719–723.

Liu, Q., Neefjes, A.C.M., Singh, A., Kobylinska, R., Mugford, S.T., Marzo, M., Canham, J., Schuster, M., van der Hoorn, R.A.L., Chen, Y., et al. (2025). Aphid effectors suppress plant immunity via recruiting defense proteins to processing bodies. Sci. Adv. **11**:eadv1447.

Liu, T.T., Yang, Q., Li, M., Zhong, B., Ran, Y., Liu, L.L., Yang, Y., Wang, Y.Y., and Shu, H.B. (2016). LSm14A plays a critical role in antiviral immune responses by regulating MITA level in a cell-specific manner. J. Immunol. **196**:5101–5111.

Lloyd, R.E. (2013). Regulation of stress granules and P-bodies during RNA virus infection. Wiley Interdiscip. Rev. RNA **4**:317–331.

Luo, Y., Na, Z., and Slavoff, S.A. (2018). P-bodies: composition, properties, and functions. Biochemistry **57**:2424–2431.

Maciej, V.D., Mateva, N., Schwarz, J., Dittmers, T., Mallick, M., Urlaub, H., and Chakrabarti, S. (2022). Intrinsically disordered regions of tristetraprolin and DCP2 directly interact to mediate decay of ARE-mRNA. Nucleic Acids Res. **50**:10665–10679.

Maldonado-Bonilla, L.D. (2014). Composition and function of P bodies in Arabidopsis thaliana. Front. Plant Sci. **5**:201.

Matheny, T., Rao, B.S., and Parker, R. (2019). Transcriptome-wide comparison of stress granules and P-bodies reveals that translation plays a major role in RNA partitioning. Mol. Cell Biol. **39**:e00313-19.

Mérai, Z., Benkovics, A.H., Nyikó, T., Debreczeny, M., Hiripi, L., Kerényi, Z., Kondorosi, É., and Silhavy, D. (2013). The late steps of plant nonsense-mediated mRNA decay. Plant J. **73**:50–62.

Merchante, C., Brumos, J., Yun, J., Hu, Q., Spencer, K.R., Enríquez, P., Binder, B.M., Heber, S., Stepanova, A.N., and Alonso, J.M. (2015). Gene-specific translation regulation mediated by the hormone-signaling molecule EIN2. Cell **163**:684–697.

Merret, R., Descombin, J., Juan, Y.T., Favory, J.J., Carpentier, M.C., Chaparro, C., Charng, Y.Y., Deragon, J.M., and Bousquet-Antonelli, C. (2013). XRN4 and LARP1 are required for a heat-triggered mRNA decay pathway involved in plant acclimation and survival during thermal stress. Cell Rep. **5**:1279–1293.

Merret, R., Nagarajan, V.K., Carpentier, M.C., Park, S., Favory, J.J., Descombin, J., Picart, C., Charng, Y.Y., Green, P.J., Deragon, J.M., et al. (2015). Heat-induced ribosome pausing triggers mRNA co-translational decay in Arabidopsis thaliana. Nucleic Acids Res. **43**:4121–4132.

Molliex, A., Temirov, J., Lee, J., Coughlin, M., Kanagaraj, A.P., Kim, H.J., Mittag, T., and Taylor, J.P. (2015). Phase separation by low complexity domains promotes stress granule assembly and drives pathological fibrillization. Cell **163**:123–133.

Motomura, K., Le, Q.T.N., Hamada, T., Kutsuna, N., Mano, S., Nishimura, M., and Watanabe, Y. (2015). Diffuse decapping

enzyme DCP2 accumulates in DCP1 foci under heat stress in Arabidopsis thaliana. Plant Cell Physiol. **56**:107–115.

Murakami, S., Olarerin-George, A.O., Liu, J.F., Zaccara, S., Hawley, B., and Jaffrey, S.R. (2025). m6A alters ribosome dynamics to initiate mRNA degradation. Cell **188**:3728–3743.e20.

Naskar, A., Nayak, A., Salaikumaran, M.R., Vishal, S.S., and Gopal, P.P. (2023). Phase separation and pathologic transitions of RNP condensates in neurons: implications for amyotrophic lateral sclerosis, frontotemporal dementia and other neurodegenerative disorders. Front. Mol. Neurosci. **16**:1242925.

Nedelsky, N.B., and Taylor, J.P. (2019). Bridging biophysics and neurology: aberrant phase transitions in neurodegenerative disease. Nat. Rev. Neurol. **15**:272–286.

Nissan, T., and Parker, R. (2008). Analyzing P-bodies in Saccharomyces cerevisiae. Methods Enzymol. **448**:507–520.

Nott, T.J., Petsalaki, E., Farber, P., Jervis, D., Fussner, E., Plochowietz, A., Craggs, T.D., Bazett-Jones, D.P., Pawson, T., Forman-Kay, J.D., et al. (2015). Phase transition of a disordered nuage protein generates environmentally responsive membraneless organelles. Mol. Cell **57**:936–947.

Olmedo, G., Guo, H., Gregory, B.D., Nourizadeh, S.D., Aguilar-Henonin, L., Li, H., An, F., Guzman, P., and Ecker, J.R. (2006). ETHYLENE-INSENSITIVE5 encodes a 5′→ 3′ exoribonuclease required for regulation of the EIN3-targeting F-box proteins EBF1/2. Proc. Natl. Acad. Sci. USA **103**:13286–13293.

Ortiz-Rodríguez, L.A., Yassine, H., Hatami, A., Nandana, V., Azaldegui, C.A., Cheng, J., Zhu, Y., Schrader, J.M., and Biteen, J.S. (2025). Stress changes the material state of a bacterial biomolecular condensate and shifts its function from mRNA decay to storage. Nat. Commun. **16**:10019.

Owen, I., and Shewmaker, F. (2019). The role of post-translational modifications in the phase transitions of intrinsically disordered proteins. Int. J. Mol. Sci. **20**:5501.

Panigrahi, G.K., Sahoo, A., and Satapathy, K.B. (2024). The processing body component varicose plays a multiplayer role toward stress management in Arabidopsis. Plant Physiol. Rep. **29**:186–192.

Parker, R., and Sheth, U. (2007). P bodies and the control of mRNA translation and degradation. Mol. Cell **25**:635–646.

Passmore, L.A., and Coller, J. (2022). Roles of mRNA poly (A) tails in regulation of eukaryotic gene expression. Nat. Rev. Mol. Cell Biol. **23**:93–106.

Perea-Resa, C., Carrasco-López, C., Catalá, R., Turečková, V., Novak, O., Zhang, W., Sieburth, L., Jiménez-Gómez, J.M., and Salinas, J. (2016). The LSM1-7 complex differentially regulates Arabidopsis tolerance to abiotic stress conditions by promoting selective mRNA decapping. Plant Cell **28**:505–520.

Perea-Resa, C., Hernández-Verdeja, T., López-Cobollo, R., del Mar Castellano, M., and Salinas, J. (2012). LSM proteins provide accurate splicing and decay of selected transcripts to ensure normal Arabidopsis development. Plant Cell **24**:4930–4947.

Pérez-Vilaró, G., Scheller, N., Saludes, V., and Díez, J. (2012). Hepatitis C virus infection alters P-body composition but is independent of P-body granules. J. Virol. **86**:8740–8749.

Petre, B., Saunders, D.G.O., Sklenar, J., Lorrain, C., Krasileva, K.V., Win, J., Duplessis, S., and Kamoun, S. (2016). Heterologous expression screens in Nicotiana benthamiana identify a candidate effector of the wheat yellow rust pathogen that associates with processing bodies. PLoS One **11**:e0149035.

Pomeranz, M.C., Hah, C., Lin, P.C., Kang, S.G., Finer, J.J., Blackshear, P.J., and Jang, J.C. (2010a). The Arabidopsis tandem zinc finger protein AtTZF1 traffics between the nucleus and cytoplasmic foci and binds both DNA and RNA. Plant Physiol. **152**:151–165.

Pomeranz, M., Lin, P.C., Finer, J., and Jang, J.C. (2010b). AtTZF gene family localizes to cytoplasmic foci. Plant Signal. Behav. **5**:190–192.

Potuschak, T., Vansiri, A., Binder, B.M., Lechner, E., Vierstra, R.D., and Genschik, P. (2006). The exoribonuclease XRN4 is a component of the ethylene response pathway in Arabidopsis. Plant Cell **18**:3047–3057.

Rao, B.S., and Parker, R. (2017). Numerous interactions act redundantly to assemble a tunable size of P bodies in Saccharomyces cerevisiae. Proc. Natl. Acad. Sci. USA **114**:E9569–E9578.

Reineke, L.C., and Lloyd, R.E. (2013). Diversion of stress granules and P-bodies during viral infection. Virology **436**:255–267.

Riggs, C.L., Kedersha, N., Ivanov, P., and Anderson, P. (2020). Mammalian stress granules and P bodies at a glance. J. Cell Sci. **133**:jcs242487.

Riyazuddin, R., Verma, R., Singh, K., Nisha, N., Keisham, M., Bhati, K.K., Kim, S.T., and Gupta, R. (2020). Ethylene: a master regulator of salinity stress tolerance in plants. Biomolecules **10**:959.

Roux, M.E., Rasmussen, M.W., Palma, K., Lolle, S., Regué, À.M., Bethke, G., Glazebrook, J., Zhang, W., Sieburth, L., Larsen, M.R., et al. (2015). The mRNA decay factor PAT1 functions in a pathway including MAP kinase 4 and immune receptor SUMM2. EMBO J. **34**:593–608.

Roy, R., and Rajyaguru, P.I. (2018). Stress granules and p-bodies: An insight into mRNA translational control and decay. Insajournal.in, 479–491.

Rymarquis, L.A., Souret, F.F., and Green, P.J. (2011). Evidence that XRN4, an Arabidopsis homolog of exoribonuclease XRN1, preferentially impacts transcripts with certain sequences or in particular functional categories. Rna **17**:501–511.

Rzeczkowski, K., Beuerlein, K., Müller, H., Dittrich-Breiholz, O., Schneider, H., Kettner-Buhrow, D., Holtmann, H., and Kracht, M. (2011). c-Jun N-terminal kinase phosphorylates DCP1a to control formation of P bodies. JCB (J. Cell Biol.) **194**:581–596.

Sahu, S., Wang, Z., Jiao, X., Gu, C., Jork, N., Wittwer, C., Li, X., Hostachy, S., Fiedler, D., Wang, H., et al. (2020). InsP7 is a small-molecule regulator of NUDT3-mediated mRNA decapping and processing-body dynamics. Proc. Natl. Acad. Sci. USA **117**:19245–19253.

Schütz, S., Nöldeke, E.R., and Sprangers, R. (2017). A synergistic network of interactions promotes the formation of in vitro processing bodies and protects mRNA against decapping. Nucleic Acids Res. **45**:6911–6922.

Sharif, H., Ozgur, S., Sharma, K., Basquin, C., Urlaub, H., and Conti, E. (2013). Structural analysis of the yeast Dhh1–Pat1 complex reveals how Dhh1 engages Pat1, Edc3 and RNA in mutually exclusive interactions. Nucleic Acids Res. **41**:8377–8390.

Sheth, U., and Parker, R. (2003). Decapping and decay of messenger RNA occur in cytoplasmic processing bodies. Science **300**:805–808.

Sheth, U., and Parker, R. (2006). Targeting of aberrant mRNAs to cytoplasmic processing bodies. Cell **125**:1095–1109.

Soma, F., Mogami, J., Yoshida, T., Abekura, M., Takahashi, F., Kidokoro, S., Mizoi, J., Shinozaki, K., and Yamaguchi-Shinozaki, K. (2017). ABA-unresponsive SnRK2 protein kinases regulate mRNA decay under osmotic stress in plants. Nat. Plants **3**:16204.

Soma, F., Takahashi, F., Suzuki, T., Shinozaki, K., and Yamaguchi-Shinozaki, K. (2020). Plant Raf-like kinases regulate the mRNA population upstream of ABA-unresponsive SnRK2 kinases under drought stress. Nat. Commun. **11**:1373.

Sorenson, R.S., Deshotel, M.J., Johnson, K., Adler, F.R., and Sieburth, L.E. (2018). Arabidopsis mRNA decay landscape arises from

specialized RNA decay substrates, decapping-mediated feedback, and redundancy. Proc. Natl. Acad. Sci. USA **115**:E1485–E1494.

Standart, N., and Weil, D. (2018). P-bodies: cytosolic droplets for coordinated mRNA storage. Trends Genet. **34**:612–626.

Steffens, A., Jaegle, B., Tresch, A., Hülskamp, M., and Jakoby, M. (2014). Processing-body movement in Arabidopsis depends on an interaction between myosins and DECAPPING PROTEIN1. Plant Physiol. **164**:1879–1892.

Sternburg, E.L., Gruijs da Silva, L.A., and Dormann, D. (2022). Post-translational modifications on RNA-binding proteins: accelerators, brakes, or passengers in neurodegeneration? Trends Biochem. Sci. **47**:6–22.

Stroberg, W., and Schnell, S. (2017). On the origin of non-membrane-bound organelles, and their physiological function. J. Theor. Biol. **434**:42–49.

Szádeczky-Kardoss, I., Gál, L., Auber, A., Taller, J., and Silhavy, D. (2018). The No-go decay system degrades plant mRNAs that contain a long A-stretch in the coding region. Plant Sci. **275**:19–27.

Teixeira, D., and Parker, R. (2007). Analysis of P-body assembly in Saccharomyces cerevisiae. Mol. Biol. Cell **18**:2274–2287.

Teixeira, D., Sheth, U., Valencia-Sanchez, M.A., Brengues, M., and Parker, R. (2005). Processing bodies require RNA for assembly and contain nontranslating mRNAs. Rna **11**:371–382.

Tharun, S., and Parker, R. (2001). Targeting an mRNA for decapping: displacement of translation factors and association of the Lsm1p–7p complex on deadenylated yeast mRNAs. Mol. Cell **8**:1075–1083.

Tosiano, M.A., Lanni, F., Mitchell, A.P., and McManus, C.J. (2025). Roles of P-body factors in Candida albicans filamentation and stress response. PLoS Genet. **21**:e1011632.

Tutucci, E., Vera, M., Biswas, J., Garcia, J., Parker, R., and Singer, R.H. (2018). An improved MS2 system for accurate reporting of the mRNA life cycle. Nat. Methods **15**:81–89.

Uniacke, J., and Zerges, W. (2008). Stress induces the assembly of RNA granules in the chloroplast of Chlamydomonas reinhardtii. J. Cell Biol. **182**:641–646.

Van Der Lee, R., Buljan, M., Lang, B., Weatheritt, R.J., Daughdrill, G.W., Dunker, A.K., Fuxreiter, M., Gough, J., Gsponer, J., Jones, D.T., et al. (2014). Classification of intrinsically disordered regions and proteins. Chem. Rev. **114**:6589–6631.

Van Leeuwen, W., and Rabouille, C. (2019). Cellular stress leads to the formation of membraneless stress assemblies in eukaryotic cells. Traffic **20**:623–638.

Vidya, E., and Duchaine, T.F. (2022). Eukaryotic mRNA decapping activation. Front. Genet. **13**:832547.

Walley, J.W., Kelley, D.R., Nestorova, G., Hirschberg, D.L., and Dehesh, K. (2010). Arabidopsis deadenylases AtCAF1a and AtCAF1b play overlapping and distinct roles in mediating environmental stress responses. Plant Physiol. **152**:866–875.

Wang, C., Schmich, F., Srivatsa, S., Weidner, J., Beerenwinkel, N., and Spang, A. (2018). Context-dependent deposition and regulation of mRNAs in P-bodies. eLife **7**:e29815.

Wang, W., Wang, C., Wang, Y., Ma, J., Wang, T., Tao, Z., Liu, P., Li, S., Hu, Y., Gu, A., et al. (2023). The P-body component DECAPPING5 and the floral repressor SISTER OF FCA regulate FLOWERING LOCUS C transcription in Arabidopsis. Plant Cell **35**:3303–3324.

Wang, Z., Yang, Q., Zhang, D., Lu, Y., Wang, Y., Pan, Y., Qiu, Y., Men, Y., Yan, W., Xiao, Z., et al. (2024). A cytoplasmic osmosensing mechanism mediated by molecular crowding–sensitive DCP5. Science **386**:eadk9067.

Weber, C., Nover, L., and Fauth, M. (2008). Plant stress granules and mRNA processing bodies are distinct from heat stress granules. Plant J. **56**:517–530.

Weick, E.M., and Lima, C.D. (2021). RNA helicases are hubs that orchestrate exosome-dependent 3′–5′ decay. Curr. Opin. Struct. Biol. **67**:86–94.

Xie, D., Chen, M., Niu, J., Wang, L., Li, Y., Fang, X., Li, P., and Qi, Y. (2021). Phase separation of SERRATE drives dicing body assembly and promotes miRNA processing in Arabidopsis. Nat. Cell Biol. **23**:32–39.

Xiong, L., Gong, Z., Rock, C.D., Subramanian, S., Guo, Y., Xu, W., Galbraith, D., and Zhu, J.K. (2001). Modulation of abscisic acid signal transduction and biosynthesis by an Sm-like protein in Arabidopsis. Dev. Cell **1**:771–781.

Xu, J., and Chua, N.H. (2009). Arabidopsis decapping 5 is required for mRNA decapping, P-body formation, and translational repression during postembryonic development. Plant Cell **21**:3270–3279.

Xu, J., and Chua, N.H. (2011). Processing bodies and plant development. Curr. Opin. Plant Biol. **14**:88–93.

Xu, J., and Chua, N.H. (2012). Dehydration stress activates Arabidopsis MPK6 to signal DCP1 phosphorylation. The EMBO journal **31**:1975–1984.

Xu, J., Yang, J.Y., Niu, Q.W., and Chua, N.H. (2006). Arabidopsis DCP2, DCP1, and VARICOSE form a decapping complex required for postembryonic development. Plant Cell **18**:3386–3398.

Xu, W., Jian, S., Li, J., Wang, Y., Zhang, M., and Xia, K. (2023). Genomic identification of CCCH-Type zinc finger protein genes reveals the role of HuTZF3 in Tolerance of Heat and Salt stress of Pitaya (Hylocereus polyrhizus). Int. J. Mol. Sci. **24**:6359.

Yoon, J.H., Choi, E.J., and Parker, R. (2010). Dcp2 phosphorylation by Ste20 modulates stress granule assembly and mRNA decay in Saccharomyces cerevisiae. J. Cell Biol. **189**:813–827.

Youn, J.Y., Dyakov, B.J.A., Zhang, J., Knight, J.D.R., Vernon, R.M., Forman-Kay, J.D., and Gingras, A.C. (2019). Properties of stress granule and P-body proteomes. Mol. Cell **76**:286–294.

Youn, J.Y., Dunham, W.H., Hong, S.J., Knight, J.D.R., Bashkurov, M., Chen, G.I., Bagci, H., Rathod, B., MacLeod, G., Eng, S.W.M., et al. (2018). High-density proximity mapping reveals the subcellular organization of mRNA-associated granules and bodies. Mol. Cell **69**:517–532.e11.

Yu, X., Li, B., Jang, G.J., Jiang, S., Jiang, D., Jang, J.C., Wu, S.H., Shan, L., and He, P. (2019). Orchestration of processing body dynamics and mRNA decay in Arabidopsis immunity. Cell Rep. **28**:2194–2205.e6.

Yu, X., Willmann, M.R., Anderson, S.J., and Gregory, B.D. (2016). Genome-wide mapping of uncapped and cleaved transcripts reveals a role for the nuclear mRNA cap-binding complex in cotranslational RNA decay in Arabidopsis. Plant Cell **28**:2385–2397.

Zeitelhofer, M., Karra, D., Macchi, P., Tolino, M., Thomas, S., Schwarz, M., Kiebler, M., and Dahm, R. (2008b). Dynamic interaction between P-bodies and transport ribonucleoprotein particles in dendrites of mature hippocampal neurons. J. Neurosci. **28**:7555–7562.

Zeitelhofer, M., Macchi, P., and Dahm, R. (2008a). Perplexing bodies: the putative roles of P-bodies in neurons. RNA Biol. **5**:244–248.

Zeke, A., Schád, É., Horváth, T., Abukhairan, R., Szabó, B., and Tantos, A. (2022). Deep structural insights into RNA-binding disordered protein regions. Wiley Interdiscip. Rev. RNA **13**:e1714.

Zhan, W., Li, Z., Zhang, J., Liu, Y., Liu, G., Li, B., Shen, R., Jiang, Y., Shang, W., Gao, S., et al. (2024). Energy stress promotes P-bodies formation via lysine-63-linked polyubiquitination of HAX1. EMBO J. **43**:2759–2788.

Zhang, R., Mao, S., and Haataja, M.P. (2023). Phase separation and gelation of chemically reactive macromolecules. Biophys. J. **122**:65a.

Zhang, X., and Guo, H. (2017). mRNA decay in plants: both quantity and quality matter. Curr. Opin. Plant Biol. **35**:138–144.

Zhang, X., Song, J., Wang, L., Yang, Z.M., and Sun, D. (2022). Identification of a DEAD-box RNA helicase BnRH6 reveals its involvement in salt stress response in rapeseed (Brassica napus). Int. J. Mol. Sci. **24**:2.

Zhang, X., Zhu, Y., Liu, X., Hong, X., Xu, Y., Zhu, P., Shen, Y., Wu, H., Ji, Y., Wen, X., et al. (2015). Suppression of endogenous gene silencing by bidirectional cytoplasmic RNA decay in Arabidopsis. Science **348**:120–123.

Zhao, L., and Kunst, L. (2016). SUPERKILLER complex components are required for the RNA exosome-mediated control of cuticular wax biosynthesis in Arabidopsis inflorescence stems. Plant Physiol. **171**:960–973.

Zheng, D., Ezzeddine, N., Chen, C.Y.A., Zhu, W., He, X., and Shyu, A.B. (2008). Deadenylation is prerequisite for P-body formation and mRNA decay in mammalian cells. J. Cell Biol. **182**:89–101.

Zhou, Y., Ćorović, M., Hoch-Kraft, P., Meiser, N., Mesitov, M., Körtel, N., Back, H., Naarmann-de Vries, I.S., Katti, K., Obrdlík, A., et al. (2024). m6A sites in the coding region trigger translation-dependent mRNA decay. Mol. Cell **84**:4576–4593.e12.

Plant Communications, Volume 7

Supplemental information

Plant P-bodies in post-transcriptional control: Composition, dynamics, and context-dependent roles

Arash Matinahmadi, Zoofa Zayani, Karolina Majewska, and Dariusz Jan Smoliński

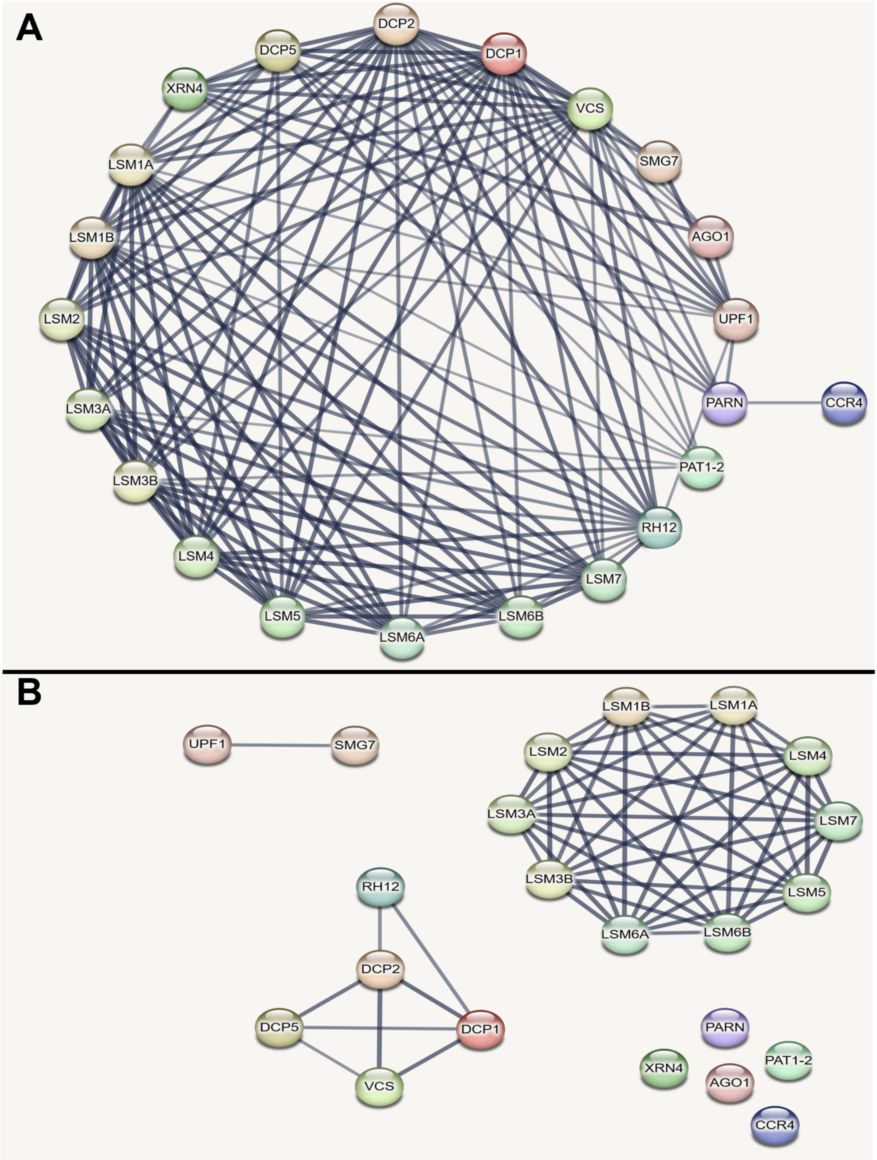
A
DCP2
DCP5
DCP1
XRN4
VCS
LSM1A
SMG7
LSM1B
AGO1
LSM2
UPF1
LSM3A
PARN
CCR4
PAT1-2
LSM3B
RH12
LSM4
LSM7
LSM5
LSM6B
LSM6A
B
LSM1B
LSM1A
UPF1
SMG7
LSM2
LSM4
LSM3A
LSM7
RH12
LSM3B
LSM5
LSM6A
LSM6B
DCP2
DCP5
DCP1
PARN
PAT1-2
XRN4
AGO1
VCS
CCR4
